# Caught in the H_inact_: Crystal Structure and Spectroscopy Reveal a Sulfur Bound to the Active Site of an O_2_‐stable State of [FeFe] Hydrogenase

**DOI:** 10.1002/anie.202005208

**Published:** 2020-07-23

**Authors:** Patricia Rodríguez‐Maciá, Lisa M. Galle, Ragnar Bjornsson, Christian Lorent, Ingo Zebger, Yoshitaka Yoda, Stephen P. Cramer, Serena DeBeer, Ingrid Span, James A. Birrell

**Affiliations:** ^1^ Department of Inorganic Spectroscopy Max Planck Institute for Chemical Energy Conversion Stiftstraße 34–36 45470 Mülheim an der Ruhr Germany; ^2^ Inorganic Chemistry Laboratory Department of Chemistry University of Oxford South Parks Road Oxford OX1 3QR UK; ^3^ Physikalische Biologie Heinrich-Heine-Universität Düsseldorf Universitätsstraße 1 40225 Düsseldorf Germany; ^4^ Physikalische Chemie/ Biophysikalische Chemie Institut für Chemie Technische Universität Berlin Straße des 17. Juni 135 10623 Berlin Germany; ^5^ Japanese Synchrotron Radiation Institute, Spring-8 1-1-1 Kouto, Mikazuki-cho Sayo-gun Hyogo 679-5198 Japan; ^6^ SETI Institute 189 Bernardo Avenue Mountain View California 94043 USA

**Keywords:** hydrogenase, protein structures, sulfide, vibrational spectroscopy, *x*-ray absorption spectroscopy

## Abstract

[FeFe] hydrogenases are the most active H_2_ converting catalysts in nature, but their extreme oxygen sensitivity limits their use in technological applications. The [FeFe] hydrogenases from sulfate reducing bacteria can be purified in an O_2_‐stable state called H_inact_. To date, the structure and mechanism of formation of H_inact_ remain unknown. Our 1.65 Å crystal structure of this state reveals a sulfur ligand bound to the open coordination site. Furthermore, in‐depth spectroscopic characterization by X‐ray absorption spectroscopy (XAS), nuclear resonance vibrational spectroscopy (NRVS), resonance Raman (RR) spectroscopy and infrared (IR) spectroscopy, together with hybrid quantum mechanical and molecular mechanical (QM/MM) calculations, provide detailed chemical insight into the H_inact_ state and its mechanism of formation. This may facilitate the design of O_2_‐stable hydrogenases and molecular catalysts.

## Graphical abstract

**Caught in the H_inact_**: [FeFe] hydrogenases are highly efficient catalysts for the interconversion of H_2_ and H^+^. However, they are highly O_2_ sensitive. Here, the X‐ray crystal structure of an O_2_‐stable state of [FeFe] hydrogenase, along with comprehensive spectroscopic analysis and molecular calculations reveals the presence of a sulfur ligand bound to the active site. These results provide crucial insight into O_2_ stability mechanisms in proteins.

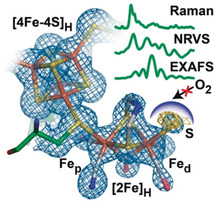

## Introduction

Hydrogen is a promising green energy carrier for the future because it can easily be produced by water electrolysis using renewable energy and, later used in a fuel cell to generate energy producing only water as a byproduct.^1^ Currently, H_2_ is produced mostly from fossil fuels or to a small extent by water electrolysis using expensive noble metal catalysts. In nature, efficient and reversible H_2_ conversion is performed by a group of metalloenzymes called hydrogenases.^2^ These biocatalysts use earth abundant metals such as nickel and/or iron in their active site.^3^ Of the three groups of hydrogenases ([NiFe] hydrogenases, [FeFe] hydrogenases and [Fe] hydrogenases), the [FeFe] hydrogenases are the most active (100 000 s^−1^ in H_2_ oxidation and up to 10 000 s^−1^ in H^+^ reduction).^4^ However, these enzymes are extremely oxygen sensitive.^5^ Vigorous efforts have been made in order to protect [FeFe] hydrogenases, and hydrogenases in general, from oxygen.^6^ Although various oxygen inactivation mechanisms have been proposed,^7^ there is still a lack of understanding on how exactly O_2_ attacks their active site. Such insights may help in designing strategies to protect hydrogenases and molecular catalysts from O_2_ damage.

The active site of the [FeFe] hydrogenases, the H‐cluster, consists of a binuclear [2Fe] sub‐cluster ([2Fe]_H_) covalently attached by a cysteine sulfur to a [4Fe–4S] cluster ([4Fe–4S]_H_).^8^ [2Fe]_H_ contains two irons bridged by the thiol groups of an aza‐propane 1,3‐dithiolate (ADT) ligand,^9^ a bridging CO ligand, with an additional CN^−^ and CO ligated to each iron. The (proximal) iron (Fe_p_) directly bound to the [4Fe–4S]_H_ sub‐cluster is always coordinatively saturated, while the distal iron (Fe_d_) possesses an open coordination site in most catalytic states, where substrates (H_2_ and H^+^) and inhibitors (including CO and O_2_) can bind. The nitrogen atom in the ADT bridge serves as a base and Fe_d_ acts as a Lewis acid, together forming a frustrated Lewis pair, which is essential to heterolytically split H_2_ at Fe_d_.^3^ The catalytic cycle of these enzymes has been extensively studied through different spectroscopic techniques.^10^

When purified aerobically from the native organism, the [FeFe] hydrogenase from *Desulfovibrio desulfuricans* remains in an inactive oxygen‐stable state called H_inact_ (or H_ox_^air^), which can be reactivated upon reduction.^4^, ^11^ This state is thought to be “overoxidized” with an Fe^II^Fe^II^ configuration at the binuclear site and an additional ligand bound to Fe_d_.^11^ The reduction of H_inact_ to an intermediate state H_trans_ is reversible while the further conversion of H_trans_ to H_ox_ is thought to be irreversible, involving the release of the putative ligand from Fe_d_.^11^ The nature of this putative ligand in the H_inact_ state has remained a mystery for more than two decades. Despite considerable spectroscopic analysis,^12^ new approaches are clearly needed to define the electronic and geometric configuration of the H‐cluster, and identify the nature of the exogenous ligand. Theoretical calculations have suggested that the extra ligand could be H_2_O or OH^−^.^13^ Interestingly, an [FeFe] hydrogenase from *Clostridium beijerinckii* has been shown to convert into the H_inact_ state in a highly reversible fashion, but the presence of an extra ligand bound in this state is so far unknown and its formation mechanism remains elusive.^14^

Recently, we showed that the H_inact_ state is formed upon oxidation of *Dd*HydAB in the presence of sulfide (Na_2_S). Based on this result, we suggested that the extra ligand bound to the open coordination site might be a sulfur species, possibly SH^−^.^15^ However, we were unable to identify whether sulfide was directly bound to the H‐cluster, in what configuration, and whether there were any additional changes to the enzyme during H_inact_ formation. In this work, we identify the nature of the additional ligand as SH^−^ through combined crystallographic and spectroscopic investigations. These results together with hybrid QM/MM calculations provide deeper understanding on the formation mechanism of this state and how it is protected against O_2_. This new insight may allow the general protection of metalloenzymes against oxygen, enabling their implementation in fuel cells and ultimately, it may provide design principles for developing O_2_‐stable bio‐inspired molecular catalysts.

## Results and Discussion

### Crystal Structure of DdHydAB in the H_inact_ State

*Dd*HydAB in the H_inact_ state was crystallized under aerobic conditions at 12 °C. Brown crystals (indicating the presence of iron–sulfur clusters) were observed within three days and retained their dark color for at least two weeks. IR spectra of crystals taken from the same drop confirmed that the *Dd*HydAB was in the H_inact_ state (Figure S1 in the Supporting Information). *Dd*HydAB in the H_inact_ state crystallized in an orthorhombic space group *P*2_1_2_1_2_1_, and the asymmetric unit contains one biological assembly. In contrast, the previously reported structure was obtained from crystals with the space group P2_1_2_1_2 and the asymmetric unit contained two biological assemblies. The structure of H_inact_ was solved using molecular replacement with the structure published by Nicolet et al.8b (PDB ID 1HFE) as a starting model, and was refined to a resolution of 1.65 Å (crystal parameters and refinement statistics in Table S1). The structure by Nicolet et al. is the only available structure of *Dd*HydAB and the redox state of the enzyme in these crystals was not defined but assumed to be a mixture of the H_ox_ and H_red_ states.

**Figure 1:**
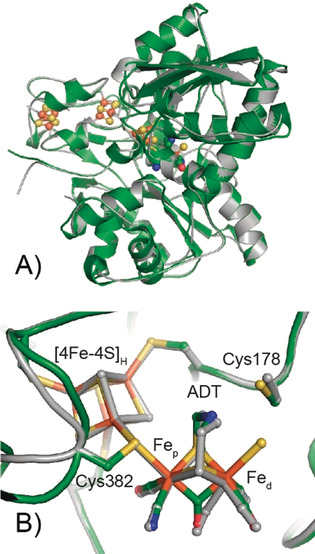
Superposition of the whole structure (A) and the active site H‐cluster region (B) of *Dd*HydAB in the H_inact_ state (green) and a partially reduced state from PDB ID 1 HFE^[[8b]^(gray). The H‐cluster, cluster ligating cysteines and C178 are shown in the sticks representation, and the protein backbone is shown in the cartoon representation. The overall architecture of *Dd*HydAB in both states is virtually identical with an RMSD of 0.631 Å (calculated for all Cα atoms of residues 2–397).

**Figure 2:**
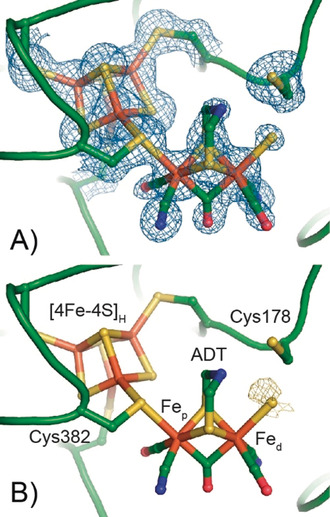
Crystal structure around the H‐cluster in the H_inact_ state of *Dd*HydAB. (A) The protein backbone is presented in the cartoon representation (green), and amino acid side chains and the H‐cluster are shown in the stick representation. A 2*Fo* − *Fc* electron density map (blue mesh, contoured at 1.0 σ) is shown for Cys 178, all Cys ligating the [4Fe–4S]_H_ sub‐cluster and the H‐cluster. An omit map generated from a model lacking the [2Fe]_H_ sub‐site and additional S ligand is shown in Figure S2. (B) Anomalous difference map (yellow mesh, contoured at 2.0 σ) is shown for the additional ligand at the apical position on Fe_d_.

The overall architecture of *Dd*HydAB in the H_inact_ state is essentially identical to the starting model with a root mean square deviation (RMSD) of 0.631 Å (calculated for all Cα atoms of residues 2–397 without outlier rejection, Figure 1 A). The electron density for the H‐cluster in the active site is well‐defined (Figure 2 A); however, the occupancy of the [2Fe] sub‐cluster had to be reduced to 0.6 to fit the experimental data. The low [2Fe] content indicates the presence of some apo protein in the preparation, partly a limitation of the artificial maturation procedure (see methods in the Supporting Information),^16^ and partly from some decomposition of the H‐cluster.^15^ A more detailed analysis of the atomic coordinates at the [2Fe] sub‐site shows that there are a few small differences in the atomic positions, in particular at the bridging ligands, ADT and CO (Figure 1 B), which likely, arise from the restraints introduced by the ligand library (crystallographic information file).^17^

The more pronounced difference in the location and orientation of the bridging CO also arises from the different ligand restraints. While Nicolet et al. modelled the CO ligand as non‐bonded; we used the ligand library also employed by Duan et al. for the [FeFe] hydrogenase from *Clostridium pasteurianum* (*Cp*HydA1).^18^ Notably, the temperature values of the bridging CO in our structure are lower than in the one reported by Nicolet et al., in agreement with our structure being that of a unique state, while Nicolet's structure was suggested to be a mixture of oxidized and reduced active states.8b No significant oxidative damage during crystallization or radiation damage to the accessory iron–sulfur clusters during the measurement were observed (Figure S3).

The electron density map reveals that the H‐cluster is intact and contains two CN^−^ ligands, two terminal CO ligands, the bridging ADT ligand, as well as the bridging CO ligand (Figure 2 A). In addition, the electron density in the active site clearly shows the presence of an additional ligand in the apical position on Fe_d_ at a distance of 2.4 Å (see Figure S4). The ligand consists of only one non‐hydrogen atom, in agreement with the presence of a (hydro)sulfide, hydroxide or oxo ligand. Modelling of the sulfur ligand with the same occupancy as that of the [2Fe] subcluster (0.6) resulted in a good fit, but with a high B‐factor (45 Å^2^), indicating some intrinsic disorder of the exogenous ligand. Modelling with an oxygen ligand gave a similar occupancy, but a slightly lower B‐factor (38 Å^2^). Thus, anomalous scattering on exactly the same crystal was utilized to provide further information about the nature of the additional ligand. By measuring diffraction data at 6 keV, the anomalous signal from the iron atoms is suppressed, while that from other heavy atoms (such as S or Cl) is enhanced. The resulting anomalous electron density map shows clear evidence for anomalous density at the apical position on Fe_d_ (Figure 2 B and Figure S5 A and B). To further support this observation, anomalous diffraction measurements were also performed before native data collection on a second crystal obtained under identical conditions (see Supporting Information, Table S1). The anomalous density map of the second crystal also showed distinct anomalous density at the apical position on Fe_d_. While this strongly supports that the additional ligand is actually a sulfur species, we cannot exclude the possibility of a Cl^−^ ligand. Interestingly, Cl^−^ has been suggested to bind to the H‐cluster under certain circumstances.^19^ Hence, we investigated the spectroscopic properties of the H_inact_ state to provide further insight.

### Characterization of the H_inact_ State by X‐Ray Absorption Spectroscopy

**Figure 3:**
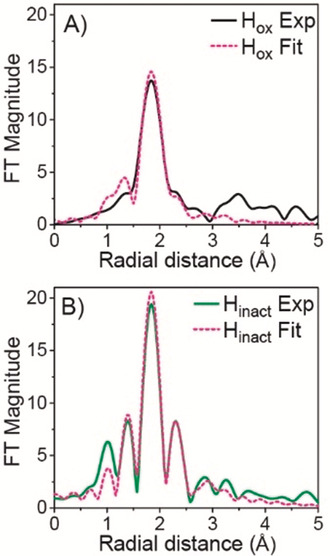
Non phase‐shifted Fourier transform of k^3^‐EXAFS of *Dd*HydAB for H_ox_ (black solid line) and H_inact_ (green solid line). Corresponding fits shown as pink dashed lines. See Figure S12 for data before Fourier transformation and Table S4 and S5 for fitting parameters.

X‐ray absorption spectroscopy (XAS) on the H_inact_ state (containing the additional ligand) and the well‐characterized H_ox_ state (lacking the additional ligand) were measured for comparison. Figure 3 shows the Fourier‐transformed (FT) spectrum of the extended X‐ray absorption fine structure (EXAFS) region for H_ox_ (Figure 3 A) and H_inact_ (Figure 3 B) after subtraction of the [4Fe–4S] cluster contribution (see discussion in the Supporting Information). The presented data thus correspond to the average environment around the two iron atoms of the H‐cluster. Comparison of the FTs clearly shows that the H‐cluster of H_inact_ has greater amplitude than that of H_ox_, consistent with the presence of an additional heavy scatterer in the first coordination sphere of H_inact_. H_ox_ is best fit with 3 Fe−C scatterers at 1.80 Å (from the terminal CN, terminal CO and the bridging CO), 2.5 Fe−S scatterers at 2.26 Å (from ADT ligand sulfurs and the Fe_p_‐bound cysteine sulfur), and an Fe−Fe scattering path at 2.60 Å (Table S4). In addition, Fe−C−O/N multiple scattering paths have to be included in the fit. The Fe−C−O/N multiple scattering paths and the Fe−Fe scattering path are highly correlated, resulting in a somewhat larger error in the fit of the Fe−Fe distance.

**Figure 4:**
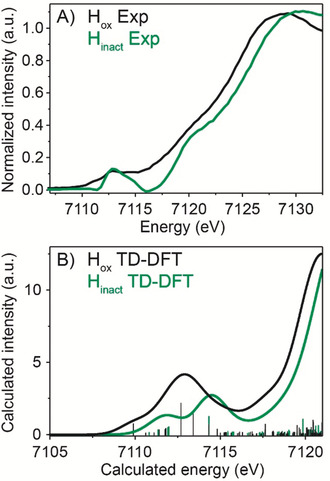
Fe K‐edge XAS experimental and calculated spectra. Fe K‐edge XAS spectra were measured in the partial fluorescence yield mode on 3 mm *Dd*HydAB samples at 10 K. (A) Fe K‐edge XAS difference spectra of *Dd*HydAB H_ox_ (black trace) and H_inact_ (green trace) both apo subtracted. (B) TD‐DFT calculated Fe K‐edge XAS spectra using the QM/MM H_ox_ model (black trace) and the H_inact_‐SH model (green trace), applying a 2 eV (FWHM) broadening and an energy shift of 30.2 eV.

For H_inact_, the first shell consists of the same scattering paths (Fe−C, Fe−S, Fe−Fe and Fe−C−O multiple scattering) with the same degeneracy for every path as for H_ox_ except for the Fe−S path, for which the degeneracy needed to be increased to N=3 (see EXAFS Discussion and Table S5 in the Supporting Information). This is consistent with the presence of an additional S ligand in H_inact_ coordinated to one of the H−cluster Fe atoms. Attempts to separate the Fe−S contributions into shorter and longer Fe−S distances (as observed in the crystal structure) resulted in the fit paths coalescing to the same distance. This suggests that the separation of the two sulfur contributions is beyond the resolution of our data (≈0.16 Å). The ability to fit unique Fe−S contributions is further complicate by the strong correlation of the various scattering paths in our system. Further, we note that similar to the protein crystallography, the EXAFS cannot distinguish between Cl or S as the additional ligand. Nevertheless, the first shell distances extracted from the EXAFS fits are in reasonable agreement, within the associated errors (≈0.1 Å), with the crystal structure (Table S5). For various crystal structures obtained with similar resolution diffraction data errors in the positions of the atoms, and hence bond lengths also, of up to 0.1 Å have been determined.^20^ Figure 4 A presents the Fe K‐edge XAS spectra of *Dd*HydAB in the H_ox_ and H_inact_ states (with the [4Fe–4S] cluster contribution subtracted, see Supporting Information for details). The shift of the rising edge toward higher energy for H_inact_ is consistent with a more oxidized binuclear site (homovalent Fe^II^Fe^II^ in H_inact_ vs. mixed‐valent Fe^I^Fe^II^ in H_ox_). The differences in the pre‐edge region (7110–7115 eV) suggest a different coordination environment of the [2Fe] sub‐cluster in the two states.

To understand the features in the experimental XAS spectra and to gain insight into the origins of the observed changes, time dependent density functional theory (TDDFT) calculations were performed with H_ox_ and H_inact_‐SH quantum mechanics/molecular mechanics (QM/MM) models (see Supporting Information Figure S7). The TDDFT calculated pre‐edge spectra (Figure 4 B) reproduce the general experimental trends in terms of the pre‐edge energies, intensity distributions and the onset of the rising edge. This indicates that the QM/MM models are consistent with the XAS data. Overall, these results support an oxidized Fe^II^Fe^II^ sub‐cluster for H_inact_ with a bound ligand at Fe_d_. We note, however, that the XAS edges are not sensitive to the exact nature of the apical ligand (see Supporting Information).

### Characterization of the H_inact_ State by Vibrational Spectroscopy

**Figure 5:**
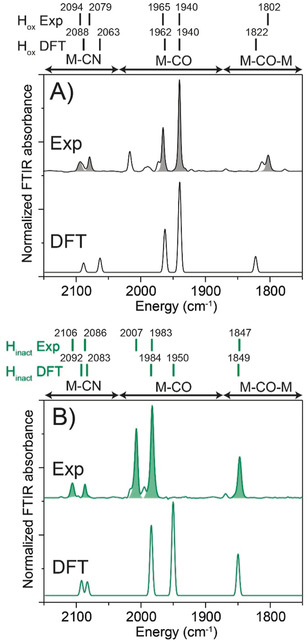
Experimental (above) and calculated (below) IR spectra of *Dd*HydAB in (A) the H_ox_ state and (B) the H_inact_ state. The experimental IR spectra were measured at 25 °C and 2 cm^−1^ resolution. For the calculated IR spectra, the QM/MM H_ox_ and H_inact_‐SH models were used. The experimental intensities were used in the plotted DFT spectra. Scaling factors for calculated frequencies were used (see Supporting Information).

Figure 5 shows experimental and calculated IR spectra of the H_ox_ and H_inact_ states. In the experimental spectra, all the bands of H_inact_ are shifted toward higher energy with respect to H_ox_. This is consistent with a more oxidized [2Fe] sub‐cluster in H_inact_, which leads to reduced backbonding into the π* orbitals of the ligands resulting in shorter CO and CN bonds.^21^ The calculated IR spectra for the H_ox_ and H_inact_‐SH QM/MM models show that the calculated frequencies are in reasonable agreement (see Supporting Information for more details). The magnitudes of the shifts are underestimated, especially for the terminal CO groups, suggesting that the experimental change in back‐bonding upon oxidation is not quite reproduced by the calculations (even though the H_inact_‐SH model is oxidized). Importantly, the calculated shift of the bridging CO, which should be sensitive to the addition of a new ligand is consistent with the experimental shift, albeit slightly underestimated as well.

**Figure 6:**
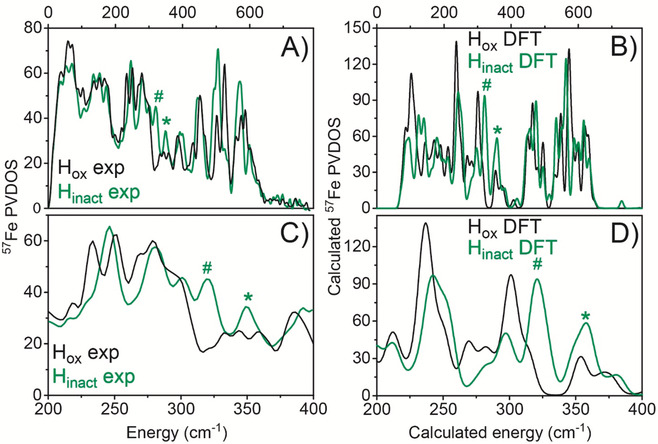
Experimental and calculated NRVS data of *Dd*HydAB in the H_ox_ and H_inact_ states. The spectra were measured on 3 mm *Dd*HydAB samples at 40–70 K. (A) Experimental NRVS data of H_ox_ (black trace) and H_inact_ (green trace). (B) Calculated NRVS data using the H_ox_ QM/MM model (black trace) and the H_inact_‐SH QM/MM model (green trace). C and D are enlargements of the Fe−S vibrational region of A and B, respectively. The asterisk indicates the peak assigned primarily to a terminal −SH group in H_inact_ while the hashtag indicates other Fe−S modes (associated with ADT ligand and Fe_p_‐Cys) that shift upon oxidation.

Nuclear resonance vibrational spectroscopy (NRVS) measures vibrational sidebands coupled to nuclear transitions for Mössbauer‐active nuclei, including ^57^Fe.^22^ NRVS has already been used to study states of [FeFe] hydrogenases, including the catalytic intermediate H_hyd_.^23^ Artificial maturation with ^57^Fe‐labelled [2Fe] precursors results in selectively labeled [2^57^Fe] sub‐clusters.^24^ As such, predominantly vibrations associated with the [2Fe] sub‐cluster are observed. Figure 6 A presents the experimental NRVS spectra of H_ox_ and H_inact_, where clear differences can be observed. Low energy features in the 150–400 cm^−1^ region emerge primarily from Fe−S vibrations (bending and stretching motions). Bands around 450 cm^−1^ are mostly due to Fe−CN motion, while the strong bands between 500–600 cm^−1^ arise predominantly from Fe−CO bending and stretching modes. By correlating the experimental spectra to QM/MM NRVS calculations, the most important differences in the spectra can be interpreted. The calculated NRVS spectra (B) using the H_ox_ and H_inact_‐SH models correlate well with the experimental. Plots C and D in Figure 6 highlight the Fe−S region of the NRVS spectra. Compelling evidence for an extra sulfur bound to H_inact_ arise from the peak at ≈350 cm^−1^ (356.52 cm^−1^ in the calculated spectrum) in the Fe−S region (marked with an asterisk). We note that the absolute prediction of complete NRVS spectra from theoretical calculations is a challenge due to the densely populated spectra and the nature of the low‐energy modes involved, which are sensitive to the computational model. It is, therefore, advantageous to focus on the difference between H_ox_ and H_inact_ and on the Fe−S region.

Calculations reveal that the increased intensity in this region of the H_inact_‐SH model (compared to H_ox_) arises from the Fe−S stretching vibration associated with an SH ligand. This feature is reproduced by the H_inact_‐SH model but cannot be reproduced in models with lighter ligands such as OH^−^ (see Supporting Information, Figure S23). The calculations cannot exclude a Cl^−^ ligand bound to Fe_d_ due to its similar mass and covalency, which gives a comparable spectrum in this region (see Supporting Information, Figure S23). However, the H_inact_ state can be formed in the strict absence of chloride (Figure S20), suggesting that a Cl^−^ bound to the open coordination site is unlikely. Furthermore, the experimental peak at ≈322 cm^−1^ (324 cm^−1^ in the calculated spectra) in H_inact_ (marked with a hashtag) is assigned to Fe−S modes from the ADT ligand and cysteine, which are shifted to higher energy (compared to H_ox_) due to a more oxidized [2Fe] sub‐cluster. The calculations demonstrate the sensitivity of NRVS spectra with respect to cluster oxidation state, but also with respect to the light vs. heavy atom nature of the ligand.

**Figure 7:**
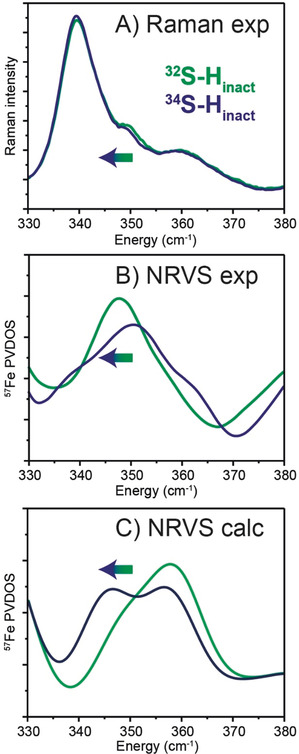
^32^S/^34^S isotope related shifts in the vibrational spectra of the H_inact_ state. A) Resonance Raman spectra were measured on 3 mm *Dd*HydAB samples at 80 K using 514 nm excitation. Experimental spectra of H_inact_ prepared with natural abundance Na_2_S (green trace) and ^34^S‐labelled Na_2_S (blue) in the Fe−S region between 330 and 380 cm^−1^ (see Figure S25 for the full spectrum). The spectra are normalized to modes at 622 cm^−1^ and 644 cm^−1^ corresponding to the amino acid side chains phenylalanine and tyrosine, respectively.^30^ Experimental (B) and QM/MM‐calculated ^57^Fe NRVS data (C) The corresponding spectra of *Dd*HydAB in the H_inact_ state with ^32^S (green) and ^34^S (blue) ligand are also displayed in the region between 330 and 380 cm^−1^ (see Figure S24 for the full spectra and a wider view of the Fe−S region). Calculated band positions using the H_inact_‐SH QM/MM model with ^32^S and ^34^S bound at open coordination site of Fe_d_ are presented in Table S12 and shown as Movies S1 and S2.

In order to directly identify the exogenous sulfur ligand, we compared NRVS spectra of H_inact_ samples prepared using natural abundance (95 % ^32^S) and ^34^S‐labelled sodium sulfide. We observed small differences between the ^32^S and ^34^S spectra, particularly in the 340–360 cm^−1^ region (Figure 7 B and Figure S24). Similar results were obtained with resonance Raman (RR) spectroscopy (Figure 7 A and Figure S25), where small changes can be observed in the 340–360 cm^−1^ region when going from the ^32^S spectrum to the ^34^S spectrum, coinciding with the expected lower frequencies of vibrations related to a heavier atom. Calculations support the assignment of two peaks in this region to Fe−S stretching modes from exogenously bound SH^−^ (Figure 7 C and Table S12).

### Mechanism of H_inact_ Formation and Implications

Using our QM/MM model, we calculated the binding of H_2_S to the H_ox_ state, (see Scheme in Figure S26).^15^ Sulfur likely reaches the H‐cluster via diffusion through the same hydrophobic gas channels used by H_2_, CO and O_2_. Thus, protonation to H_2_S (p*K*_a_≈7)^25^ in solution facilitates this process. Interestingly, partial formation of a very similar H_inact_ state with Na_2_Se could be achieved (Figure S27), but only at pH 4, where the enzyme is not very stable. This supports the idea that the neutral species (H_2_S or H_2_Se) are involved, as H_2_Se has a much lower p*K*_a_ (3.89)^25^ than H_2_S. Direct H_2_S binding to H_ox_ is calculated to be thermoneutral (Δ*G*=+0.2 kcal mol^−1^), while deprotonation of bound H_2_S by the NH group of the ADT is quite favorable (Δ*G*=−4.9 kcal mol^−1^). This leads to an Fe−SH bound intermediate that is favorable with respect to free H_2_S (Δ*G*=−4.7 kcal mol^−1^). The H‐cluster is subsequently oxidized to give an Fe^II^Fe^II^ binuclear sub‐cluster, a process driven by application of oxidizing potentials. This likely proceeds via proton‐coupled electronic rearrangement, whereby the electron is first transferred from the binuclear sub‐cluster to the [4Fe–4S] sub‐cluster, followed by its oxidation. The oxidation steps take place by one‐electron transfers from the [4Fe–4S]_H_ subcluster, via the F‐clusters, to the available high potential oxidant (including oxygen). Calculations performed with our H_inact_‐OH QM/MM model suggest that H_2_O binds effectively at H_ox_ but will not deprotonate via the ADT ligand, due to the larger p*K*_a_ difference than for H_2_S (see Supporting Information).

A number of other metalloenzymes bind sulfide under similar conditions. Both [NiFe] hydrogenase and Ni‐dependent CO dehydrogenase are inhibited by sulfide at high applied potentials, presumably by sulfide binding in a bridging position between Ni and Fe.^26^ Recent studies suggest that, in nitrogenase, reductive displacement of an active site “belt” sulfide could be important for binding of substrates/inhibitors.^27^ Thus, binding of additional sulfides to compensate for increased positive charge on oxidized metal ions could be a common theme among enzymes, highlighting the importance of understanding how sulfide interacts with metals in nature. Diiron model complexes including thioether groups are also involved in oxidation state dependent sulfur coordination from the S group.^28^ However, in none of these cases has additional oxygen protection due to sulfur binding been demonstrated, as is observed in [FeFe] hydrogenase.

Handling air‐sensitive enzymes such as [FeFe] hydrogenases under air has definite advantages, particularly, for crystallization and manipulation of crystals. Our *Dd*HydAB H_inact_ structure is the first [FeFe] hydrogenase structure for which the redox state has been defined using single crystal spectroscopy, as previously demonstrated for [NiFe] hydrogenases.^29^ This provides the opportunity to directly correlate structural and spectroscopic properties of the H‐cluster. Interestingly, there are very few differences in the structure of the H‐cluster compared with previously published structures, suggesting an H‐cluster environment that minimizes redox state dependent structural changes, lowering reorganization energy and enhancing catalysis. Air‐stable [FeFe] hydrogenases may also be industrially useful for example, in fuel cells. Fuel cells containing [FeFe] hydrogenase embedded in a redox polymer have been prepared under strict anaerobic conditions,^6^ but could be prepared under air with the H_inact_ state, simplifying the process and increasing the scalability.

### The Relevance of O_2_ Protection via H_inact_ In Vivo

The bacterium *Desulfovibrio desulfuricans* has evolved in anaerobic environments and, therefore, its hydrogenase is extremely oxygen sensitive, becoming inactivated irreversibly even by traces of O_2_.^11^, ^31^ Although the mechanism of oxygen inactivation is not yet completely understood, O_2_ is believed to attack the active site by binding to the open coordination site on Fe_d_.^30^ If the bound‐O_2_ cannot be reduced to water, it may form reactive oxygen species, which could destroy the active site. As the open coordination site is blocked by sulfide in the H_inact_ state, this prevents O_2_ binding and destruction of the active site.

A plausible scenario is that in vivo, *Dd*HydAB is constantly exposed to H_2_S (since *Desulfovibrio desulfuricans* reduces sulfate to sulfide). Under reductive conditions H_2_S is displaced by H_2_, binding to the H‐cluster. In the presence of oxygen, however, H_2_S becomes locked to the H‐cluster forming the H_inact_ state to protect the enzyme from oxygen inactivation. It is interesting that the [FeFe] hydrogenase from *Clostridium beijerinckii* (*Cb*H5A) can form the H_inact_ state without exogenous sulfide.^14^ How, the H_inact_ state in this enzyme differs structurally from that in *Dd*HydAB is not known, but it seems likely that, in the absence of available sulfide in this organism, an endogenous sulfur ligand, such as a cysteine nearby the active site, has evolved to play a role. Another important difference between these two enzymes is that *Dd*HydAB functions as a periplasmic H_2_ uptake enzyme with extremely high activity,^32^ while *Cb*H5A shows a strong bias for H_2_ production.^14^ This lack of activity in H_2_ uptake may be due to spontaneous formation of H_inact_ at high potentials. As *Dd*HydAB requires exogenous sulfide, it will only be inactive when sulfide levels are high, which may serve to regulate metabolism.

## Conclusion

Here, we provide direct structural and spectroscopic evidence for an exogenously bound sulfur in the apical coordination site of the [FeFe] hydrogenase from *Desulfovibrio desulfuricans* in the O_2_‐stable H_inact_ state. In our previous work, we showed that exogenous sulfide was required for H_inact_ formation, but we were unable to demonstrate if and how sulfide binds to the H‐cluster. The 1.65 Å crystal structure shows electron density at the apical position on the distal Fe and anomalous diffraction suggests this is consistent with sulfur. EXAFS shows an additional sulfur in the Fe‐coordination environment of H_inact_ compared with H_ox_. Fe K‐edge XAS data reveal a more oxidized [2Fe] sub‐cluster in H_inact_ compared to H_ox_ and a different coordination environment of the Fe ions in the [2Fe] subcluster. Comparison of H_inact_ and H_ox_ NRVS spectra, as well as ^32^S/^34^S isotope‐labelling in both NRVS and resonance Raman spectroscopy, provide additional compelling evidence for an exogenous sulfur ligand. Calculations with an H_inact_‐SH model provide close agreement to all the experimental data and shed light on the mechanism of forming H_inact_. In particular, the most likely pathway involved H_2_S binding at the open coordination site, followed by proton transfer via the ADT ligand to the proton transfer channel. H_inact_ formation is then completed upon proton coupled electronic reconfiguration of the H‐cluster and oxidation of [4Fe–4S]_H_. Since we previously demonstrated that this in vitro H_inact_ approach works for other [FeFe] hydrogenases such as *Chlamydomonas reinhardtii* (*Cr*HydA1), it demonstrates the wider applicability of this method. Thus, it would be interesting to perform similar structural and spectroscopic studies of H_inact_ in other enzymes, including *Cb*H5A and *Cr*HydA1. Our highly complementary structural, spectroscopic and theoretical approach represents a major advance for the understanding the function of this O_2_‐stable state and its mechanism of formation, as well as a possible implementation of these enzymes in biotechnological applications.

## Conflict of interest

The authors declare no conflict of interest.

## Supporting information

As a service to our authors and readers, this journal provides supporting information supplied by the authors. Such materials are peer reviewed and may be re‐organized for online delivery, but are not copy‐edited or typeset. Technical support issues arising from supporting information (other than missing files) should be addressed to the authors.

Supplementary

Supplementary

Supplementary

Supplementary

## References

[1] N. Armaroli , V. Balzani , ChemSusChem 2011, 4, 21–36. 21226208 10.1002/cssc.201000182

[2] K. A. Vincent , A. Parkin , F. A. Armstrong , Chem. Rev. 2007, 107, 4366–4413. 17845060 10.1021/cr050191u

[3] W. Lubitz , H. Ogata , O. Rüdiger , E. Reijerse , Chem. Rev. 2014, 114, 4081–4148. 24655035 10.1021/cr4005814

[4] W. G. Martin , B. R. Glick , S. M. Martin , Can. J. Microbiol. 1980, 26, 1214–1223. 7006765 10.1139/m80-203

[5] F. A. Armstrong , J. Hirst , Proc. Natl. Acad. Sci. USA 2011, 108, 14049–14054. 21844379 10.1073/pnas.1103697108 PMC3161523

[6] A. A. Oughli , F. Conzuelo , M. Winkler , T. Happe , W. Lubitz , W. Schuhmann , O. Rüdiger , N. Plumeré , Angew. Chem. Int. Ed. 2015, 54, 12329–12333; 10.1002/anie.201502776 26073322

[7a] S. T. Stripp , G. Goldet , C. Brandmayr , O. Sanganas , K. A. Vincent , M. Haumann , F. A. Armstrong , T. Happe , Proc. Natl. Acad. Sci. USA 2009, 106, 17331–17336; 19805068 10.1073/pnas.0905343106 PMC2765078

[7b] K. D. Swanson , M. W. Ratzloff , D. W. Mulder , J. H. Artz , S. Ghose , A. Hoffman , S. White , O. A. Zadvornyy , J. B. Broderick , B. Bothner , P. W. King , J. W. Peters , J. Am. Chem. Soc. 2015, 137, 1809–1816; 25579778 10.1021/ja510169s

[7c] A. Kubas , C. Orain , D. De Sancho , L. Saujet , M. Sensi , C. Gauquelin , I. Meynial-Salles , P. Soucaille , H. Bottin , C. Baffert , V. Fourmond , R. B. Best , J. Blumberger , C. Léger , Nat. Chem. 2017, 9, 88–95. 27995927 10.1038/nchem.2592 PMC5597964

[8a] J. W. Peters , W. N. Lanzilotta , B. J. Lemon , L. C. Seefeldt , Science 1998, 282, 1853–1858; 9836629 10.1126/science.282.5395.1853

[8b] Y. Nicolet , C. Piras , P. Legrand , C. E. Hatchikian , J. C. Fontecilla-Camps , Structure 1999, 7, 13–23. 10368269 10.1016/s0969-2126(99)80005-7

[9] A. Silakov , B. Wenk , E. Reijerse , W. Lubitz , Phys. Chem. Chem. Phys. 2009, 11, 6592–6599. 19639134 10.1039/b905841a

[10a] A. Adamska , A. Silakov , C. Lambertz , O. Rüdiger , T. Happe , E. Reijerse , W. Lubitz , Angew. Chem. Int. Ed. 2012, 51, 11458–11462; 10.1002/anie.201204800 23109267

[10b] C. Sommer , A. Adamska-Venkatesh , K. Pawlak , J. A. Birrell , O. Rüdiger , E. J. Reijerse , W. Lubitz , J. Am. Chem. Soc. 2017, 139, 1440–1443; 28075576 10.1021/jacs.6b12636

[10c] D. W. Mulder , Y. Guo , M. W. Ratzloff , P. W. King , J. Am. Chem. Soc. 2017, 139, 83–86; 27973768 10.1021/jacs.6b11409

[10d] S. Katz , J. Noth , M. Horch , H. S. Shafaat , T. Happe , P. Hildebrandt , I. Zebger , Chem. Sci. 2016, 7, 6746–6752. 28451119 10.1039/c6sc01098a PMC5355867

[11] W. Roseboom , A. L. de Lacey , V. M. Fernández , C. Hatchikian , S. P. J. Albracht , J. Biol. Inorg. Chem. 2006, 11, 102–118. 16323019 10.1007/s00775-005-0040-2

[12] A. S. Pereira , P. Tavares , I. Moura , J. J. G. Moura , B. H. Huynh , J. Am. Chem. Soc. 2001, 123, 2771–2782. 11456963 10.1021/ja003176+

[13] C. Greco , M. Bruschi , L. De Gioia , U. Ryde , Inorg. Chem. 2007, 46, 5911–5921. 17602468 10.1021/ic062320a

[14] S. Morra , M. Arizzi , F. Valetti , G. Gilardi , Biochemistry 2016, 55, 5897–5900. 27749036 10.1021/acs.biochem.6b00780

[15] P. Rodríguez-Maciá , E. J. Reijerse , M. van Gastel , S. DeBeer , W. Lubitz , O. Rüdiger , J. A. Birrell , J. Am. Chem. Soc. 2018, 140, 9346–9350. 30008217 10.1021/jacs.8b04339

[16] J. A. Birrell , K. Wrede , K. Pawlak , P. Rodriguez-Maciá , O. Rüdiger , E. J. Reijerse , W. Lubitz , Isr. J. Chem. 2016, 56, 852–863.

[17a] I. D. Brown , B. McMahon , Acta Crystallogr. Sect. B 2002, 58, 317–324; 12037350 10.1107/s0108768102003464

[17b] S. R. Hall , F. H. Allen , I. D. Brown , Acta Crystallogr. Sect. A 1991, 47, 655–685.

[18] J. Duan , M. Senger , J. Esselborn , V. Engelbrecht , F. Wittkamp , U.-P. Apfel , E. Hofmann , S. T. Stripp , T. Happe , M. Winkler , Nat. Commun. 2018, 9, 4726. 30413719 10.1038/s41467-018-07140-x PMC6226526

[19] M. del Barrio , M. Sensi , L. Fradale , M. Bruschi , C. Greco , L. de Gioia , L. Bertini , V. Fourmond , C. Léger , J. Am. Chem. Soc. 2018, 140, 5485–5492. 29590528 10.1021/jacs.8b01414

[20a] J. M. Guss , H. D. Bartunik , H. C. Freeman , Acta Crystallogr. Sect. B 1992, 48, 790–811; 1492962 10.1107/s0108768192004270

[20b] E. I. Solomon , R. K. Szilagyi , S. DeBeer George , L. Basumallick , Chem. Rev. 2004, 104, 419–458. 14871131 10.1021/cr0206317

[21a] J. F. Siebel , A. Adamska-Venkatesh , K. Weber , S. Rumpel , E. Reijerse , W. Lubitz , Biochemistry 2015, 54, 1474–1483; 25633077 10.1021/bi501391d

[21b] P. Rodríguez-Maciá , E. Reijerse , W. Lubitz , J. A. Birrell , O. Rüdiger , J. Phys. Chem. Lett. 2017, 8, 3834–3839. 28759237 10.1021/acs.jpclett.7b01608

[22] H. Wang , E. E. Alp , Y. Yoda , S. P. Cramer , in Metalloproteins: Methods and Protocols (Eds.: J. C. Fontecilla-Camps, Y. Nicolet), Humana Press, Totowa, NJ, 2014, pp. 125–137.

[23] V. Pelmenschikov , J. A. Birrell , C. C. Pham , N. Mishra , H. Wang , C. Sommer , E. Reijerse , C. P. Richers , K. Tamasaku , Y. Yoda , T. B. Rauchfuss , W. Lubitz , S. P. Cramer , J. Am. Chem. Soc. 2017, 139, 16894–16902. 29054130 10.1021/jacs.7b09751 PMC5699932

[24] R. Gilbert-Wilson , J. F. Siebel , A. Adamska-Venkatesh , C. C. Pham , E. Reijerse , H. Wang , S. P. Cramer , W. Lubitz , T. B. Rauchfuss , J. Am. Chem. Soc. 2015, 137, 8998–9005. 26091969 10.1021/jacs.5b03270 PMC4799848

[25] J. Bjerrum , L. G. Sillén , G. K. Schwarzenbach , C. Berecki-Biedermann , Stability constants of metal-ion complexes, with solubility products of inorganic substances. 2 : Inorganic ligands, Chemical society, London, 1958.

[26a] K. A. Vincent , J. A. Cracknell , J. R. Clark , M. Ludwig , O. Lenz , B. Friedrich , F. A. Armstrong , Chem. Commun. 2006, 5033–5035; 10.1039/b614272a 17146518

[26b] V. C. C. Wang , M. Can , E. Pierce , S. W. Ragsdale , F. A. Armstrong , J. Am. Chem. Soc. 2013, 135, 2198–2206. 23368960 10.1021/ja308493k PMC3894609

[27a] D. Sippel , M. Rohde , J. Netzer , C. Trncik , J. Gies , K. Grunau , I. Djurdjevic , L. Decamps , S. L. A. Andrade , O. Einsle , Science 2018, 359, 1484–1489; 29599235 10.1126/science.aar2765

[27b] T. Spatzal , K. A. Perez , O. Einsle , J. B. Howard , D. C. Rees , Science 2014, 345, 1620–1623. 25258081 10.1126/science.1256679 PMC4205161

[28] M. Razavet , S. J. Borg , S. J. George , S. P. Best , S. A. Fairhurst , C. J. Pickett , Chem. Commun. 2002, 700–701. 10.1039/b111613b 12132485

[29a] Y. Ilina , C. Lorent , S. Katz , J.-H. Jeoung , S. Shima , M. Horch , I. Zebger , H. Dobbek , Angew. Chem. Int. Ed. 2019, 58, 18710–18714; 10.1002/anie.201908258 PMC6916344 31591784

[29b] E. Siebert , Y. Rippers , S. Frielingsdorf , J. Fritsch , A. Schmidt , J. Kalms , S. Katz , O. Lenz , P. Scheerer , L. Paasche , V. Pelmenschikov , U. Kuhlmann , M. A. Mroginski , I. Zebger , P. Hildebrandt , J. Phys. Chem. B 2015, 119, 13785–13796. 26201814 10.1021/acs.jpcb.5b04119

[30] F. M. B. Paulo , T. C. Freire , J. A. Lima , F. E. A. Melo , J. M. Filho , in Raman Spectroscopy and Applications, Vol. Open access peer-reviewed Edited Volume, IntechOpen, 2017.

[31] K. A. Vincent , A. Parkin , O. Lenz , S. P. J. Albracht , J. C. Fontecilla-Camps , R. Cammack , B. Friedrich , F. A. Armstrong , J. Am. Chem. Soc. 2005, 127, 18179–18189. 16366571 10.1021/ja055160v

[32] E. C. Hatchikian , N. Forget , V. M. Fernandez , R. Williams , R. Cammack , Eur. J. Biochem. 1992, 209, 357–365. 1327776 10.1111/j.1432-1033.1992.tb17297.x

