# Supplementary material for: Caught in the Hinact: Crystal Structure and Spectroscopy Reveal a Sulfur Bound to the Active Site of an O2‐stable State of [FeFe] Hydrogenase
Source: Angew Chem Int Ed Engl. 2020 Jul 23;59(38):16786–94. doi: 10.1002/anie.202005208 (PMC7540559; doi:10.1002/anie.202005208)
Supplement: Supplementary file 2 — Supplementary [file ANIE-59-16786-s002.pdf]

Angewandte Chemie
Eine Zeitschrift der Gesellschaft Deutscher Chemiker

Supporting Information

Caught in the H_{inact}: Crystal Structure and Spectroscopy Reveal a Sulfur Bound to the Active Site of an O_2-stable State of [FeFe] Hydrogenase

Patricia Rodríguez-Maciá[+], Lisa M. Galle[+], Ragnar Bjornsson[+], Christian Lorent, Ingo Zebger, Yoshitaka Yoda, Stephen P. Cramer, Serena DeBeer, Ingrid Span,* and James A. Birrell**

anie_202005208_sm_miscellaneous_information.pdf
anie_202005208_sm_miscellaneous_information.cif
anie_202005208_sm_Movie_S1.mpeg
anie_202005208_sm_Movie_S2.mpeg

This PDF file includes:

Material and Methods
Supplementary Text
Figs. S1 to S27
Tables S1 to S12
Captions for Movies S1 to S2
Supplementary References

Other Supplementary Information for this manuscript includes the following:

Movie files S1 and S2.
XYZ files for H_{ox}, H_{inact}-OH, H_{inact}-SH, H_{inact}-Cl QM/MM models.

Materials and Methods

Preparation of samples.

Preparation of the H_{inact} state was performed as described previously.[1] *Dd*HydAB was produced recombinantly in *E. coli* BL21 (DE3) ΔiscR cells as a fusion protein with a Strep-II tag on the C-terminus of the large subunit. Cells were grown aerobically and expression was induced anaerobically. Cells were harvested anaerobically in a glovebox containing 2% H_2 in N_2, broken by sonication and the protein was purified using Strep-tag affinity chromatography. $(Et_4N)_2[Fe_2(adt)(CO)_4(CN)_2]$ ($[2Fe]^{adt}$) and $(Et_4N)_2[^{57}Fe_2(adt)(CO)_4(CN)_2]$ ($[2^{57}Fe]^{adt}$) were synthesized following published procedures.[2] Artificial maturation was carried out by mixing 1 mM $[2Fe]^{adt}$ or $[2^{57}Fe]^{adt}$ with 0.2 mM *Dd*HydAB for 50 h at 35 ^oC. Excess $[2Fe]^{adt}$ was removed on a PD-10 desalting column and *Dd*HydAB by shaken at 15 ^oC in the dark until the IR spectrum was devoid of the H_{ox}-CO state, typically 60 h. We note that even after this long maturation time, there is still some apo protein remaining in the preparation. $Na_2{}^{34}S$ was prepared by reduction of elemental ^{34}S with sodium metal using a naphthalene catalyst. For preparation of the H_{inact} state, samples were transferred to a 100% N_2 glovebox, diluted to 0.2 mM, oxidized with 1 mM hexaammineruthenium (III) chloride (HAR), mixed with 10 mM Na_2S or $Na_2{}^{34}S$, followed by 10 mM HAR. Following this, samples were stirred under air, precipitate was removed on 100 kDa MWCO concentrators, salts were removed on PD-10 columns, samples were concentrated on 30 kDa MWCO concentrators, and samples were frozen at -80 ^oC until use.

X-ray crystallography.

*Dd*HydAB was exchanged into 10 mM Tris-HCl pH 7.6 for crystallization. Crystals were obtained using sitting drop vapor diffusion with 1:1 protein solution (15 mg/mL) to reservoir solution (0.9 M lithium chloride, 26% polyethylene glycol 6000, 0.1 M sodium acetate) at 12 °C. Crystals were observed within 3 days, flash-frozen after soaking for 60 s in cryo-protectant (50% (w/v) aqueous polyethylene glycol 6000), mounted into MiTeGen MicroMounts/MicroLoops, and stored in liquid nitrogen. Datasets were collected at Deutsches Elektronensynchrotron (DESY, Hamburg, Germany) beamline P11, PETRAIII at 100 K. A high-resolution dataset for structure refinement was collected at 12.4 keV. For the anomalous scattering contribution of sulfur atoms, a second, high-multiplicity data set was collected at 6 keV. Data were processed using XDS.[3]

Molecular replacement and structure refinement were performed using CCP4[4] with PDB ID: 1HFE as a starting model. Data reduction was performed with AIMLESS,[5] phasing with Phaser,[6] Coot for model building, adding water molecules to the model, and model validation, and REFMAC[7] for refinement. The final model contained 97.71% in the favored region and 0.00% in outlier regions of the Ramachandran plot as defined by MolProbity.[8] Maps were calculated using FFT[9] and CMAPCOEFF.[4] The omit map was calculated with REFMAC using a model lacking the [2Fe] subcluster and additional S ligand. PyMOL was used to prepare the figures and to calculate the root-mean-square deviation (RMSD) of the Cα atoms of residues 2-397 using the align command with the number of cycles set to 0, thus, not including outlier rejection. The atomic coordinates have been deposited with the Protein Data Bank, Research Collaboratory for Structural Bioinformatics at Rutgers University (PDB ID: 6SG2).

Single crystal infrared spectroscopy.

Crystals from the same drop used for X-ray crystallography were collected, placed on homemade MgF_2 plates and immediately frozen in liquid N_2. IR spectra were recorded using a Bruker Tensor 27 FT-IR spectrometer linked to a Hyperion 3000 IR microscope equipped with a 20× IR transmission objective and a mercury cadmium telluride (MCT) detector as previously described.[10] The temperature was set to -40 °C by a liquid-N_2-cooled cryo-stage (Linkam Scientific instruments). Data were processed using Bruker OPUS software.

X-ray absorption spectroscopy.

PFY Fe K-edge XAS data were recorded at Stanford Synchrotron Radiation Laboratory (SSRL, California, USA) at beamline 9-3 using a 100-element solid state Ge detector (Canberra) with a SPEAR storage ring current of ~500 mA at a power of 3.0 GeV as already described.[11] The utilized beam flux was ~3 x 10^{10}

photons/s and the beam size was about 1 (height) × 4 (width) mm^2. Incoming X-rays were selected using a Si(220) double-crystal monochromator. A Rh-coated mirror was used for harmonic rejection. Samples were kept at ~10 K in a liquid helium flow cryostat. Data were calibrated using an iron foil, with the first inflection point set to 7111.2 eV. Spectra were collected on various spots in the sample with a scan time of 27 min/spot. No damage was observed within 30 min collection on a single spot. Final data analysis was performed with scans showing no radiation damage. All PFY-EXAFS and PFY-XANES spectra shown in the main text and here are averages of 6 to 8 scans and the final H_{ox} and H_{inact} spectra presented in all the cases are the apo subtracted spectra. Data were processed using Athena.[12] Spectra were normalized in energy (eV) space, by fitting a third-order polynomial to the pre-edge region and subtracted throughout the entire EXAFS spectrum. A three-region cubic spline (with the AUTOBK function within Athena) was employed to model the background function to a minimum of k = 16 Å^{-1} for all spectra. Fourier transformation (FT) was performed over a windowed k range indicated in the figure captions, and all FT spectra are shown without phase shift correction. Theoretical EXAFS fittings were calculated using Artemis[12] by using the multiple scattering FEFF6 code.[13] ADT, H_{ox} and H_{inact} models were generated from the ADT crystal structure,[2c] the PDB crystal structure of H_{ox} (PDB ID 1HFE)[14] and our resolved H_{inact} crystal structure (PDB: 6SG2). EXAFS scattering paths were calculated with FEFF6[12] and employed the FEFF cards: SIG2 0.001; RMAX 5; NLEGS 4. The Fourier-transform spectrum of each were fit over a R range of 1 – 3 Å (non-phase shift corrected). The FT arises from a transform of k^3-weighted EXAFS spectrum with a Hann window of k = 2 to 12 Å^{-1} for H_{ox}, k = 2.5 to 12.5 Å^{-1} for H_{inact} and k = 2 to 12.717 Å^{-1} for ADT. A single ΔE_0 variable was used for all paths in a given fit. S_0^2 was set to 0.9 for all the fits. The E_0 for ADT, H_{ox} and H_{inact} was set to 7118.210 eV. The quality (goodness) of the final fits was examined by their R-value.

QM/MM calculations of XAS, IR and NRVS spectra

QM/MM model generation is detailed in a separate chapter below. A QM/MM numerical partial Hessian (QM-atom only displacements) for each state was calculated using DL-FIND in Chemshell.[15] NRVS partial vibrational densities of states (PVDOS) were calculated using the orca_vib program (part of ORCA)[16] and the orca_mapspc program was used to create broadened vibrational spectra (Gaussian lineshape with 12 cm^{-1} (FWHM)). XAS spectra were calculated on QM/MM-optimized geometries using TDDFT and ZORA-TPSSh. RIJCOSX was used in TDDFT calculations with a decontracted auxiliary basis set (SARC/J). Up to 400 roots were calculated using the Fe 1s orbitals as donor orbitals while the acceptor space consisted of all virtual orbitals. Electric dipole, magnetic dipole and quadrupole contributions were included. The TDDFT calculations were polarized by MM point charges. Orca_mapspc was used for spectral broadening (Gaussian lineshape with 1 eV (FWHM)).

Nuclear resonance vibrational spectroscopy.

NRVS spectra were recorded at SPring-8 BL19LXU using a Si(111) double crystal high heat load monochromator (HHLM) to produce 14.414 keV radiation with ≈1.0 eV resolution, followed by a high energy resolution monochromator (HRM) [Ge(422)x2Si(975)] to increase the resolution to ≈0.8 meV. The beam flux was ≈5.4 x 10^9 photons/s and the beam size was about 0.6 (height) × 1 (width) mm^2. A 2 x 2 avalanche photodiode (APD) detector array collected the delayed nuclear fluorescence and Kα fluorescence. The temperature at the base of the sample was maintained at 10 K with a liquid He cryostat. The Stoke/anti-Stoke imbalance derived real sample temperatures were 40-70 K. NRVS spectral analysis was performed using the PHOENIX software package executed through spectratools.[17] Energy scale calibration was achieved with $[NEt_4][^{57}FeCl_4]$.

Resonance Raman spectroscopy.

Resonance Raman spectra were collected on a LabRam HR-800 Jobin Yvon confocal Raman spectrometer connected to a liquid-N_2-cooled charge-coupled device (CCD) as previously described.[10] The 514 nm emission line of an Ar^+-ion laser with 2 mW power was used for excitation. Temperature was set to 80 K by a liquid-N_2-cooled cryo-stage (Linkam Scientific instruments). Data were processed using Bruker OPUS software.

Supplementary Text

Crystallography Analysis and Supplementary Discussion.

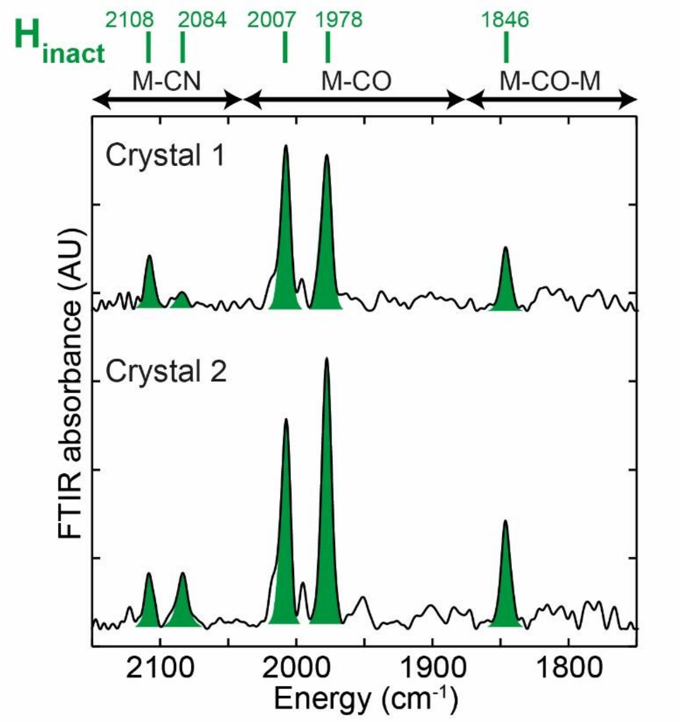

Fig. S1. IR spectra of *Dd*HydAB H_{inact} single crystals measured at -40 ºC.

To verify that the state being measured in the X-ray diffraction experiments is indeed H_{inact}, we performed IR spectroscopy on crystals from the same crystal drop used for X-ray diffraction data collection. Crystals were transferred to MgF_2 plates, as much liquid was removed as possible, and the plates were frozen in liquid nitrogen. The IR spectra of the crystals show the active site is indeed in the H_{inact} state. Differences in the relative intensities of the peaks may be related to the orientation of the anisotropic crystal in the IR beam, as the extinction coefficients are orientation dependent. Small differences of the band positions may additionally correlate with temperature induced shifts.

Table S1. Data collection and refinement statistics.

	*Dd*HydAB native[A]	*Dd*HydAB sulfur[B]	*Dd*HydAB sulfur 2[C]
**Data collection**			
Space group	$P2_12_12_1$	$P2_12_12_1$	$P2_12_12_1$
Cell dimensions			
a, *b*, *c* (Å)	49.34, 86.82, 88.17	49.28, 86.75, 88.08	49.21, 85.97, 88.14
α, β, γ (°)	90, 90, 90	90, 90, 90	90, 90, 90
Resolution (Å)	43.41-1.65 (1.68-1.65)	38.91-1.94 (1.98-1.95)	88.14 – 2.01 (2.04-2.01)
R_{merge}	0.151 (2.024)	0.106 (0.900)	0.141 (1.188)
$I / \sigma I$	10.6 (1.0)	19.5 (0.4)	14.9 (0.7)
Completeness (%)	100 (100)	85.8 (14.4)	95.7 (58.4)
Multiplicity	13.0 (12.5)	19.6 (2.1)	19.5 (3.9)
CC(1/2)	0.998 (0.526)	0.999 (0.482)	0.999 (0.386)
Anomalous completeness		81.9 (1.6)	92.4 (41.3)
Anomalous multiplicity		10.6 (1.9)	10.6 (2.4)
**Refinement**			
Resolution (Å)	43.45 – 1.65		
No. reflections	44094		
R_{work} / R_{free}	0.179/0.213		
No. atoms			
Protein	3755		
Ligand/ion	42		
Water	138		
B-factors			
Protein	24.9		
Ligand/ion	23.0		
Water	26.7		
R.m.s. deviations			
Bond lengths (Å)	0.008		
Bond angles (°)	2.112		

*Values in parentheses are for highest-resolution shell.

[A]Native dataset collected at 12 keV on an H_{inact} crystal. [B]Anomalous dataset collected at 6 keV on the same H_{inact} crystal as for A after the native data collection. [C]Additional anomalous dataset collected at 6 keV on a fresh H_{inact} crystal obtained under identical conditions.

As explained in the main text, the structure of H_{inact} was solved using molecular replacement with the structure published by Nicolet *et al.* (PDB ID 1HFE) as a starting model, and was refined to a resolution of 1.65 Å with the crystal parameters and refinement statistics shown in the Table above. The overall structure of *Dd*HydAB in the H_{inact} state is essentially identical to the starting model with a root mean square deviation (RMSD) of 0.631 Å (calculated for all Cα atoms of residues 2-397 without outlier rejection, Figure 1 of the main text). Detailed analysis of the atomic coordinates at the [2Fe] sub-site shows a few small differences in the atomic positions, in particular at the bridging ligands, ADT and CO. Most likely, these deviations arise from the restraints introduced by the ligand description. In Nicolet's structure one of the bridging ligands is modelled as a propane 1,3-dithiolate, whereas our model contains a 2-azapropane 1,3-dithiolate ligand. The differences in C-C and C-N bond angles may result in the different position of the bridgehead. This has been observed experimentally in the *Cp*HydA1 ADT vs PDT crystal structures reported by Esselborn and coworkers.[18] The more notable difference in the location and orientation of the bridging CO also arises from the different ligand restraints. While Nicolet *et al.* modelled the CO ligand as non-bonded; we used the

ligand description that was also employed by Duan *et al.* for the [FeFe] hydrogenase from *Clostridium pasteurianum* (*Cp*HydA1).[19] When taking a careful look at the omit map calculated for the model in absence of the [2Fe] sub-site and the additional S ligand, we observe that the electron density of the ligand is slightly shifted towards Fe_d, as was also observed for some of the *Cp*HydA1 crystal structures.[18]

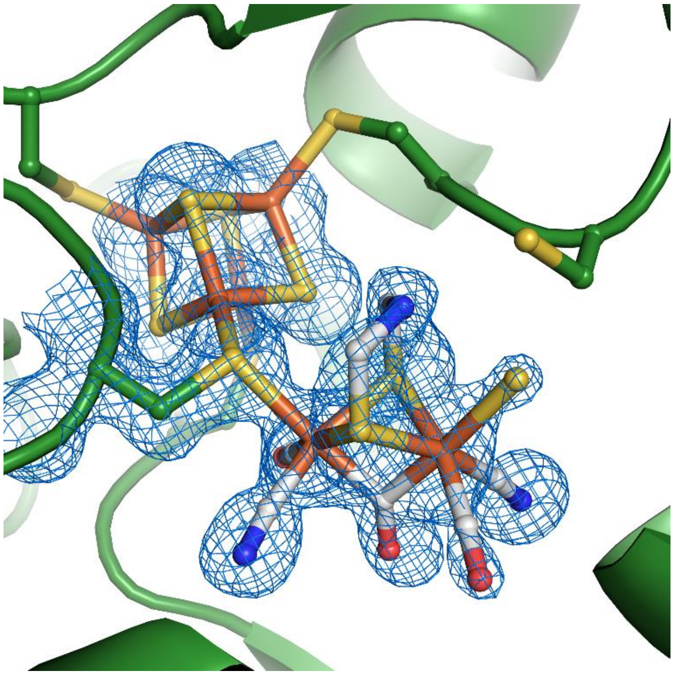

Fig. S2. Omit map of the H-cluster in the H_{inact} crystal structure. The electron density shown in blue is contoured at 1.0 σ with $2F_o$-F_c coefficients; the coordinates of the [2Fe] subsite including the additional sulfur atom have been omitted from the model for the electron density calculation.

Supplementary Discussion on Ligand Occupancy

As stated in the main text, the diiron site and exogenous ligand were both modelled with an occupancy of 0.6. This is likely due to a combination of incomplete artificial maturation of the H-cluster despite the long maturation times used, and some loss of the diiron subcluster during the 3 days of crystallization. Despite the lower [2Fe] content the electron density is well-defined and the B-factors for the [2Fe] subcluster are reasonably low (15 – 29 $Å^2$), except for the additional sulfide that has a higher B-factor (45 $Å^2$) indicating some intrinsic disorder. Most importantly, the F_O-F_C maps generated with different occupancies of the additional ligand and assuming a sulfur ligand indicate that the best fit between model and experimental data is achieved with identical occupancy for the subcluster and the additional ligand, and thus every [2Fe] subcluster has an exogenously bound ligand.

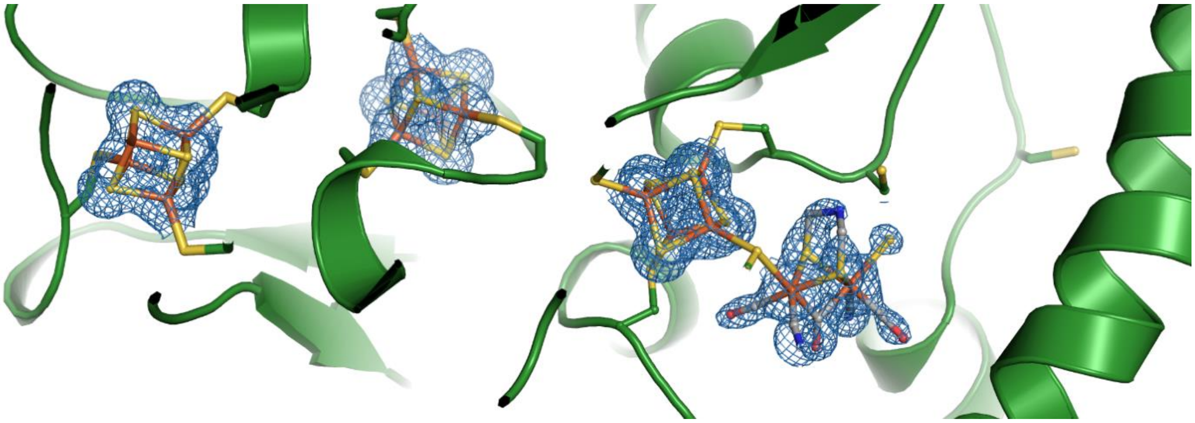

Fig. S3. Electron density map of all the cofactors in the H_{inact} crystal. The protein backbone is presented in the cartoon representation (green), and amino acid side chains and the H-cluster are shown in the stick representation. A 2Fo − Fc electron density map (blue mesh, contoured at 1.0 σ) is shown for the three [4Fe-4S] clusters and the [2Fe] subcluster. Based on the electron density of the [4Fe-4S] clusters, it does not appear that any oxidative damage to these clusters occurred during the preparation and crystallization, or radiation damage during measurement.

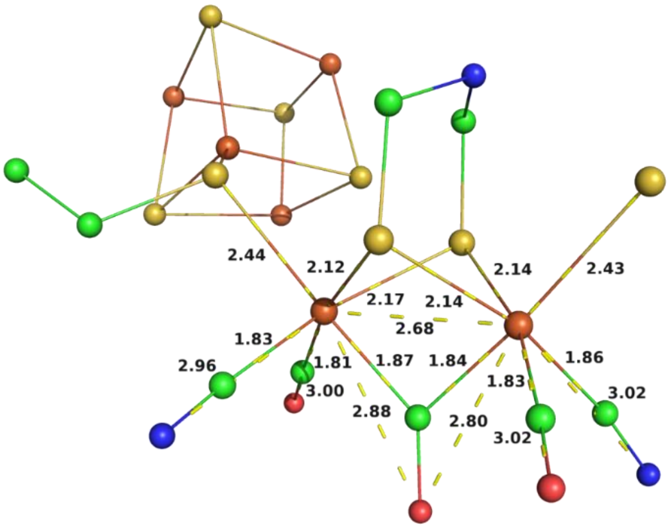

Fig. S4. H-cluster of the H_{inact} crystal structure (PDB: 6SG2) with distances.

A B

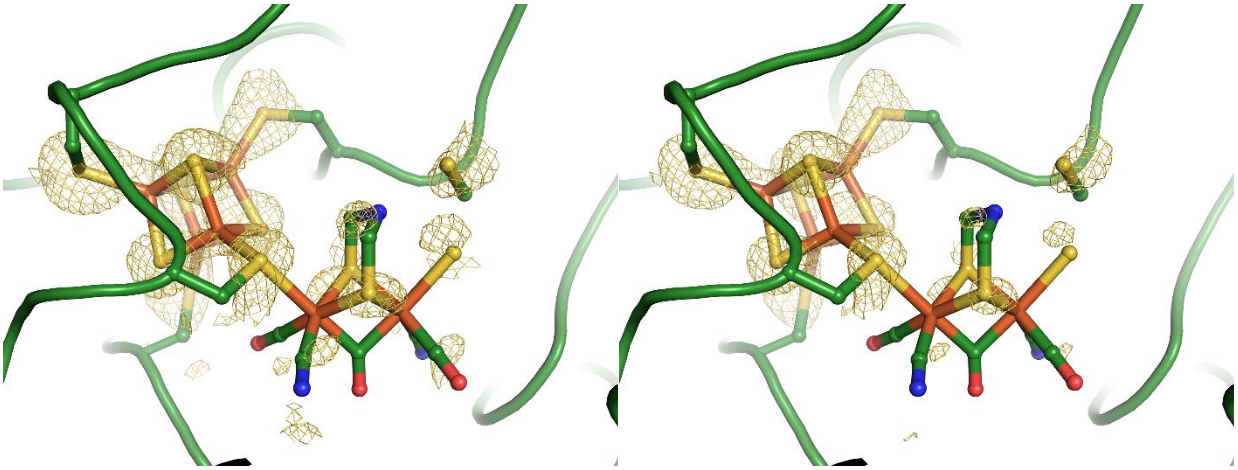

Fig. S5. Anomalous dispersion data. **A**: Anomalous density of the H-cluster from H_{inact} crystal in yellow mesh, σ = 2.0. **B**: Same data as in **A** but at σ = 2.5.

QM/MM Setup Supplementary Discussion.

Force Field Parameters:

The CHARMM36 protein force-field [20] was used in the MM preparation and the QM/MM calculations with modifications to account for the metal clusters. Non-bonded parameters for the metal clusters were derived and the metal clusters were kept fixed during all MM optimizations and MD simulations. Atomic charges for the metal clusters were derived from ZORA-TPSSh [21]/ZORA-def2-TZVP [22] calculations (including the CPCM continuum) using Hirshfeld population analysis [23]. Appropriate Lennard-Jones parameters from the CHARMM force-field were used for the clusters. The cysteines bound to the iron-sulfur clusters were modelled as deprotonated cysteines using parameters available in CHARMM36.

MM model preparation and solvation:

The whole protein was modelled classically and the initial structure was based on the crystal structure from this work. GROMACS, version 5.0.4 [24] was used to set up the original MM model and add missing hydrogens. Multiple occupancies were removed (Glu53, Met54, Met170, Cys178, Pro195, Pro319 and Leu395). Protonation states of titratable residues were determined using manual inspection of hydrogen bonding patterns. The following histidine residues (in chains L and S) were flipped: L-14, L-58, L-75, L-141, L-196, L-351 and S-89 to account for likely hydrogen-bonding to other residues. Histidines L-14, L-61, L-74, L-140, L-195, L-370, S-82, S-85, S-89, S-91 were protonated at the N_{δ} position, L-25, L-350, S-1 at N_{ε} and L-57 at both N_{δ} and N_{ε}. All aspartate and glutamate residues were modelled as deprotonated. All lysine residues were modelled as protonated.

Water molecules (as oxygen atoms) present in the crystal structure were kept and hydrogens added using GROMACS. The system was solvated by placing the protein inside a 90 x 90 x 90 Å box and filling the box with TIP3P waters. The system has a total positive charge of +7 after the hydrogenation step. To balance the charge, 7 Cl^- counter-ions were added to the solvent. The final total system size was 71491 atoms (Figure S6). The structure was optimized (metal clusters and Cys178 kept frozen) and then subjected to a 5 ns MD simulation (metal clusters and Cys178 kept frozen). Bond constraints (LINCS algorithm [25]) were applied to all bonds in order to maintain a 1 fs time-step during the simulation. A 4-chain Nosé-Hoover thermostat [26] with coupling to the whole system, was used for heating and maintaining a simulation temperature of 300 K. The system was gradually heated from 50 K to 300 K in 0-500 ps. The RMSD (with respect to crystal structure) of all heavy protein atoms converged at ~0.3 Å during the simulation.

QM/MM calculation details:

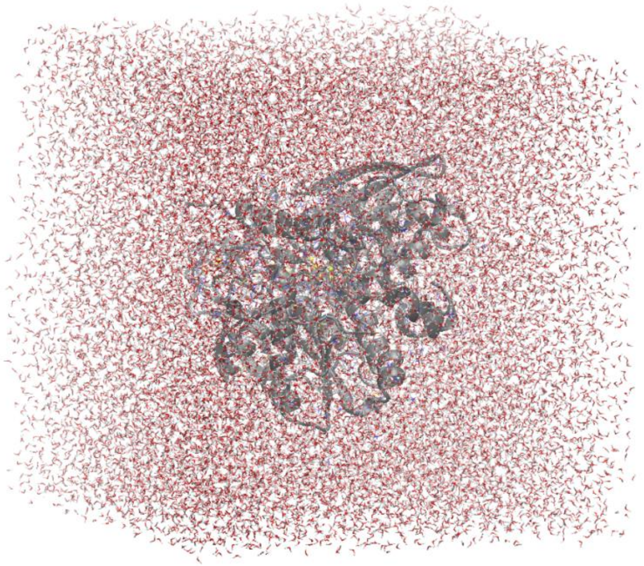

Fig. S6. The full solvated model of *Dd*HydAB.

The MM model was used directly in the QM/MM calculations (without periodic boundary conditions). Chemshell version 3.7 [15] was used for all QM/MM calculations and the system was imported using scripts previously described [27]. The ORCA quantum chemistry code (version 4.1.1) [16a] was interfaced to Chemshell via a modified Chemshell-ORCA interface. All calculations used electrostatic embedding and link atoms were used to terminate the QM-MM border together with the charge-shift procedure as implemented in Chemshell [28]. For the QM part in the QM/MM optimizations, the TPSSh hybrid density functional [29] with D3BJ dispersion correction [30] and the ZORA relativistic approximation [31] was used with the relativistically recontracted def2-TZVP basis sets [20, 32]. The RIJCOSX approximation [33] was used to speed up Coulomb and Exchange integrals. The QM region used for H-cluster calculations is shown in Figure S7. The MM part was calculated using DL_POLY [34] as implemented in Chemshell using the modified CHARMM36 force-field. The QM/MM geometry optimizations were done using the DL-FIND [35] program inside Chemshell. An active region of 1081 atoms was used (approximately spherical region around the H-cluster). The HDLC coordinate system was used in all optimizations. Numerical QM/MM frequency calculations (IR, NRVS) were performed at the same level of theory.

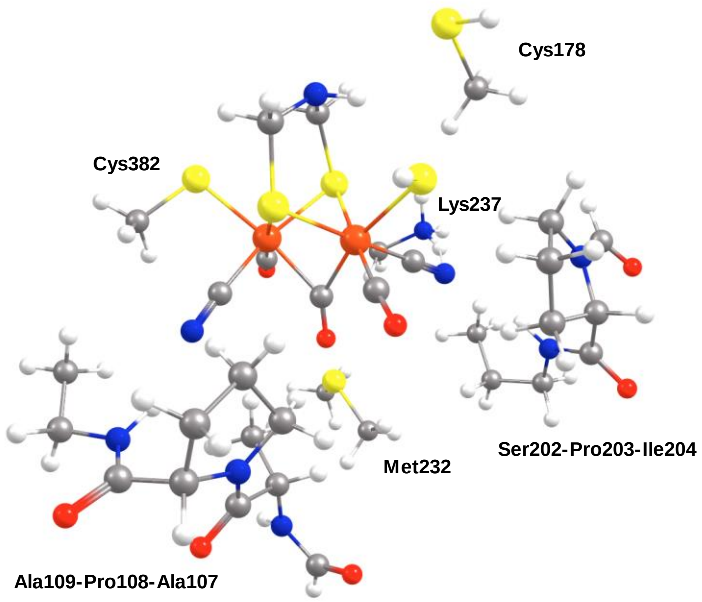

Fig. S7. The QM-region (117 atoms) used in all QM/MM calculations (link atoms are included). H_{inact}-SH model shown.

Computational models for H_{inact}

The primary models for H_{inact} that were considered (with SH, Cl or OH ligands) are shown in Figure S8 along with the model for H_{ox}. Only the [FeFe] cluster geometry is shown in Figure S8, but all calculations used for the QM-region are shown in Figure S7.

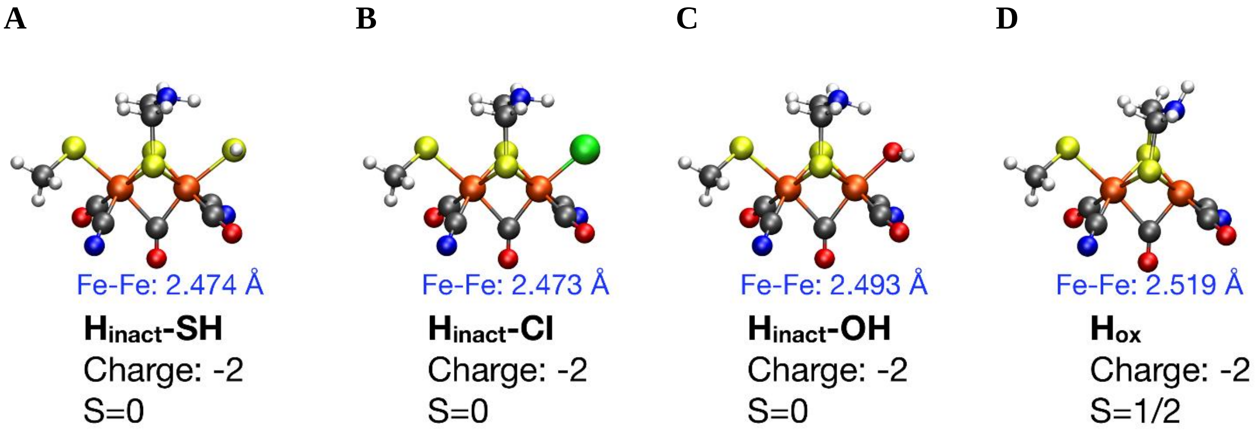

Fig. S8. Active site models of the H-cluster in H_{inact} and H_{ox} states. Geometries were QM/MM optimized using the QM-region shown in Figure S7. **A** shows the H_{inact}-SH model, **B** the H_{inact}-Cl model **C** the H_{inact}-OH model. The model for the H_{ox} state is shown in **D**. See supplementary XYZ files for the structural coordinates for each model.

A deprotonated sulfide model H_{inact}-S (charge of -3) was calculated but this gave a highly reactive sulfide that subsequently reacted with a carbonyl group, resulting in the implausible structure shown in Figure S9.

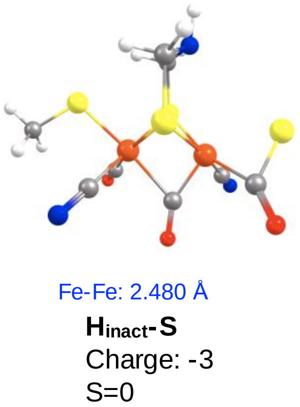

Fig. S9. Alternative model: Deprotonated H_{inact}-S. The H_{inact}-S model results in spontaneous SCO ligand formation as shown.

The H_{inact}-SH model in Figure S8 (and Figure S10 left) features the NH-group of the ADT ligand making a hydrogen bond to the SH group. This was found to be the energetically most favorable conformer (by >10 kcal/mol). Alternative conformations of the H_{inact}-SH model that were explored (including different conformations of Cys178), are shown in Figure S10. Based on the energetics (polarized QM energies) the alternative conformers are less plausible models for H_{inact} and they result in overall very similar [FeFe] structures.

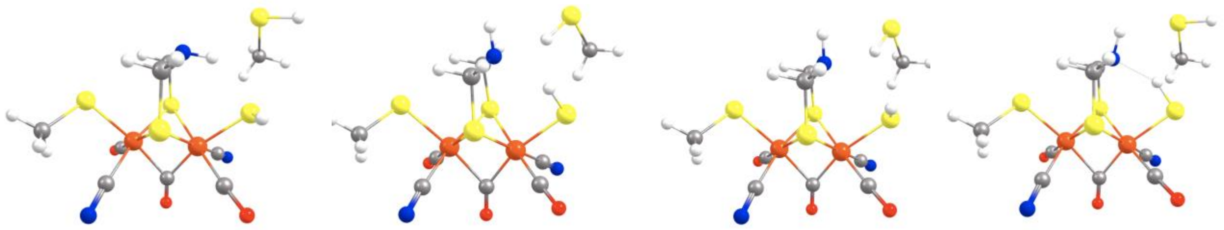

Fe-Fe: 2.474 Å	Fe-Fe: 2.474 Å	Fe-Fe: 2.473 Å	Fe-Fe: 2.478 Å
**H_{inact}-SH**	**H_{inact}-SH'-1**	**H_{inact}-SH'-2**	**H_{inact}-SH'-3**
Charge: -2	Charge: -2	Charge: -2	Charge: -2
S=0	S=0	S=0	S=0
ΔE=0.0	ΔE=11.4	ΔE=11.1	ΔE=10.3

Fig. S10. Alternative models of the H_{inact}-SH model (the hydrogen-bonding with Cys178 also shown).

EXAFS and XAS Data Analysis and Supplementary Discussion.

EXAFS and XAS Data Analysis:

*Dd*HydAB contains a total of 14 Fe ions (a [2Fe] sub-cluster and a [4Fe-4S] cluster from the H-cluster plus two accessory [4Fe-4S] clusters). XAS is a bulk technique, which measures an average of all the Fe sites in the sample. Thus, to only see changes occurring at the [2Fe] sub-cluster in the H_{inact} and H_{ox} states, the contribution of the three [4Fe-4S] clusters needed to be removed from the total spectra. Therefore, the spectrum of the apo-protein (before maturation with the [2Fe] subcluster) was measured and subtracted from the H_{inact} and H_{ox} spectra after normalization in energy (eV) space as follows:

H_{ox} apo-subtracted = H_{ox} - 12/14 x Apo

H_{inact} apo-subtracted = H_{inact} - 12/14 x Apo

For comparison, a sample of the diiron precursor complex $(Et_4N)_2[Fe_2$(2-aza-propane 1,3-dithiolate)$(CO)_4(CN)_2]$ (referred to simply as ADT) dissolved in 100 mM Tris-HCl, 150 mM NaCl, pH 8 buffer was also measured.

Figure S11A shows the raw Fe K-edge XANES spectra for the apo-enzyme, holo-H_{ox}, and holo-$H_{inact.}$ In Figure S11B the H_{inact} and H_{ox} apo-subtracted spectra can be seen with error bars included, indicating the standard deviation in the intensity over 8 scans.

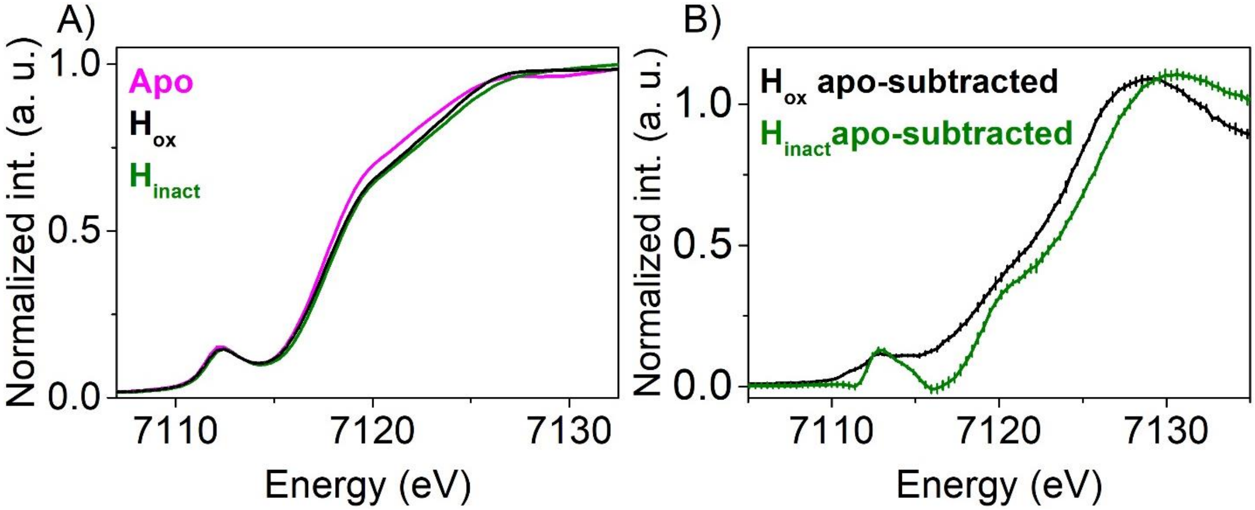

Fig. S11. Fe K-edge XAS partial fluorescence yield raw data (A) and apo-subtracted data with error bars included (B). Fe K-edge XAS were collected in the PFY-mode on 3 mM *Dd*HydAB (apo, pink trace) and 3 mM holo *Dd*HydAB samples (H_{inact} green trace and H_{ox} black trace), all measured at 10 K.

It is clear that the raw spectra of H_{ox} and H_{inact} are highly dominated by the features of the apo-enzyme, where the Fe centers have a non-centrosymmetric tetrahedral geometry allowing 3d−4p mixing, giving an intense pre-edge feature around 7112 eV. The edge energy is influenced by the sulfur ligands, which are heavy scatterers that shift the edge toward lower energies [36]. Figure S11 B shows apo-subtracted spectra, also reported in Figure 4A of the main text, but including error bars generated as reported previously [37]. This clearly indicates the significance of the differences in the pre-edge and edge regions of the XANES spectra (see Tables S2 and S3 for fitting of the pre-edge features).

Table S2. Parameters derived from the pre-edge fitting of the H_{ox} apo-subtracted spectrum. The fitting was done in blueprintXAS.

**Intensity peak**	STD	**Position peak**	STD	**HWHM peak**	STD	**Gaussian Fract peak**	STD
0.2	8.47E-17	7114.52	0.00937	1	0	0.1756	0.02994
0.25	0	7112.83	0.01826	0.99636	0.01962	0.14759	0.00373
0.06361	0.00561	7111.24	0.0886	0.93868	0.03068	0.00187	0.01026

Table S3. Parameters derived from the pre-edge fitting of the H_{inact} apo-subtracted spectrum. The fitting was done in blueprintXAS.

Intensity peak	STD	Position peak	STD	**HWHM peak**	STD	Gaussian Fract. peak	STD
0.139327	0.00694335	7114.17	0.01806	**0.834737**	0.004203	0.940279	0.084045
0.186502	0.00555292	7112.81	0.027456	**0.671056**	0.020763	0.801367	0.0028507

EXAFS Supplementary Discussion:

EXAFS fittings reported in the main text have been performed only on the H_{ox} and H_{inact} apo-subtracted spectra. The EXAFS data plotted in “k-space” (Figure S12 and insets of Figures S13, S14 and S15) are fourier transformed (FT) to give the final plots shown in Figure 3 of the main text and Figures S13, S14 and S16 of the SI. FT is the product of a transform of the k^3-weighted EXAFS spectrum with a Hann window over the range of k = 2 to 12 Å^{-1} for H_{ox} and k = 2.5 to 12.5 Å^{-1} for H_{inact}.

It is apparent from the current and previous *Dd*HydAB crystal structures of [FeFe] hydrogenases [14], that the Fe_p-S_{cys} bond is quite long (~ 2.5 Å) compared with the Fe-S_{ADT} bonds (~2.2 Å). As the EXAFS scattering intensity decreases with the square of the distance between the absorber and the scatterer, it was not clear whether inclusion of the contribution of the Fe_p-S_{cys} was necessary in the fit and in case it is, whether it would be possible to group it with the same average distance as the Fe-S_{ADT} contributions. Thus, for the H_{ox} data a set of fits were carried out in which the coordination number (degeneracy of the path, N) of the Fe-sulfur-path (Fe-S path) were altered. A degeneracy of the Fe-S path of N = 2.5 (Figure 3 A, Figure S12 A, Table S4) gave a reasonable Debye-Waller (DW or σ^2) factor (a measurement of the static and thermal disorder) of the Fe-S path. When the degeneracy of the Fe-S path was set to N = 2 (excluding the contribution from Fe_p-S_{cys}), the DW factor of the Fe-S path became negative and that of the Fe-Fe path became unrealistically small (<0.002 Å^2, see Figure S13 A and Table S6). This indicates that the contribution of Fe_p-S_{cys} is essential. A larger degeneracy of N = 3 (Figure S13 B and Table S7) gave unreasonably large DW factors.

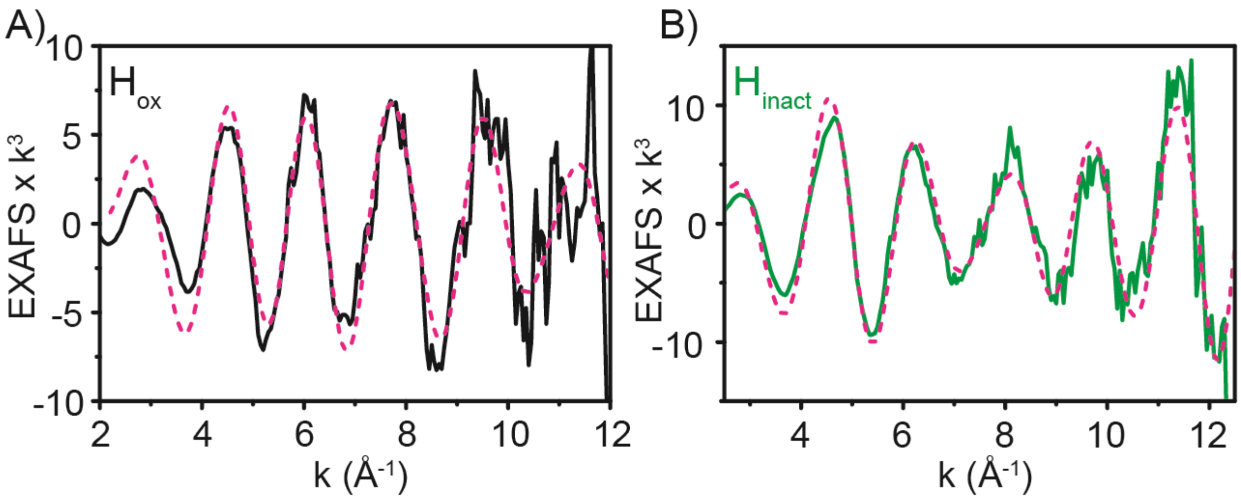

Fig. S12. k^3-EXAFS spectra of H_{ox} (A) and H_{inact} (B) corresponding to the FT of the EXAFS from Figure 3 in the main text. Fitting parameters are shown in Table S4 and S5.

Table S4. EXAFS fit parameters for Figure 3A of *Dd*HydAB in the H_{ox} state from the main text and Figure S12 A.

S_0^2= 0.9 / ΔE_0= -0.34 eV

R-factor: 0.0598

Scattering Path	N	σ² (x10⁻³ Å²)	+/-	R (Å)	+/-
**Fe-C**	3	4.74	2.9	1.800	0.023
**Fe-C-O/N**	4	5.22	4.1	2.983	0.030
**Fe-Fe**	1	1.92	2.8	2.603	0.021
**Fe-S**	**2.5**	1.44	1.2	2.264	0.009

Table S5. EXAFS fit parameters for Figure 3B of *Dd*HydAB in the H_{inact} state from the main text and Figure S12 B.

S_0^2= 0.9 / ΔE_0= 1.102 eV

R-factor: 0.0681

Scattering Path	N	σ² (x10⁻³ Å²)	+/-	R (Å)	+/-
**Fe-C**	3	3.95	3.1	1.902	0.026
**Fe-C-O/N**	4	5.96	3.8	2.984	0.032
**Fe-Fe**	1	2.00	set	2.632	set
**Fe-S**	**3**	1.00	set	2.233	0.007

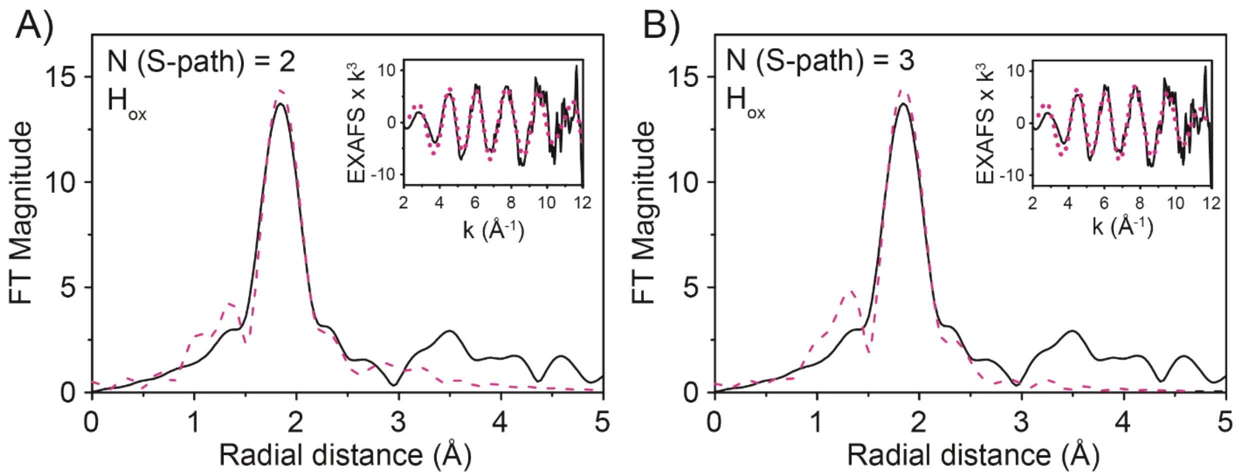

Fig. S13. k^3-EXAFS and FT spectra for the set of H_{ox} fits changing the degeneracy of the Fe-S path. **A**: decreasing the degeneracy (N) of the Fe-S path to N = 2 (Table S6). **B**: increasing the degeneracy (N) of the Fe-S path to N = 3 (Table S7).

Table S6. EXAFS fit parameters for *Dd*HydAB in the H_{ox} state with a degeneracy of the Fe-S path of N = 2 in Figure S13 A.

S_0^2= 0.9 / ΔE_0= -0.34 eV

R-factor: 0.0588

Scattering Path	N	σ² (x10⁻³ Å²)	+/-	R (Å)	+/-
**Fe-C**	3	5.77	3.3	1.800	0.025
**Fe-C-O/N**	4	3.81	3.2	2.981	0.024
**Fe-Fe**	1	0.59	2.1	2.598	0.016
**Fe-S**	**2**	-0.24	1.1	2.260	0.008

Table S7. EXAFS Fit Parameters for *Dd*HydAB in the H_{ox} state with a degeneracy of the Fe-S path of N = 3 in Figure S13 B.

S_0^2= 0.9 / ΔE_0= -0.34 eV

R-factor: 0.0687

Scattering Path	N	σ² (x10⁻³ Å²)	+/-	R (Å)	+/-
**Fe-C**	3	3.74	2.7	1.800	0.021
**Fe-C-O/N**	4	7.8	6.6	2.978	0.044
**Fe-Fe**	1	4.2	4.5	2.606	0.031
**Fe-S**	**3**	3.09	1.35	2.267	0.01

For H_{inact} EXAFS fits, when the sulfur degeneracy was set to N = 3 (Figure 3 B, Figure S12 B, Table S5) the DW factor of the Fe-S path is very small ($\sigma^2 \approx 0.001$ Å^2), suggesting that the fit is strongly dominated by the S-scattering path. Such a small DW value normally suggests that the degeneracy of the path should be even higher. When a set of fits changing the degeneracy of the Fe-S path (but fixing the DW of the Fe-Fe-path to $\sigma^2 = 0.002$ Å^2) were examined, it was clearly observed that: *1)* when the degeneracy of the Fe-S path is decreased to N = 2.5, its DW factor becomes negative and the quality of the fit (evaluated by the R-value) also decreases (Figure S14 A and Table S8), and *2)* when N = 3.5, the DW factor increases to a more reasonable value (see Figure S14 B and Table S9) but the overall quality of the fit decreases. Since the DW factor of the Fe-S path is strongly correlated with both the Fe-Fe distance and the DW factor of the Fe-Fe path, the EXAFS analysis can only give a range on the Fe-S coordination number of 3 to 3.5. Thus, while the N = 3.5 fit cannot be excluded, it is certain that N (Fe-S path) > 2.5 and, therefore, there are more sulfur scatterers in H_{inact} than H_{ox}. Similarly, and as mentioned in the main text, fits attempting to separate the Fe-S scattering paths into shorter and longer Fe-S distances (as observed in the crystal structure) resulted in the fit paths converging to the same distance. Thus, the separation of the two sulfur contributions is beyond the resolution of our data (~0.16 Å). Similar to the protein crystallography, we also note the EXAFS cannot distinguish between Cl or S as the additional ligand. Due to the strong correlation between the various parameters of the various paths, more accuracy is not possible. However, taken in the context of the crystal structure and the other spectroscopic data, the EXAFS analysis are consistent with the notion of a single additional sulfur ligand bound to the $[2Fe]_H$ subcluster in the H_{inact} state relative to H_{ox}, and the distances obtained from the EXAFS fits are in reasonable agreement, within the associated errors (~0.1 Å), with the crystal structure.[38]

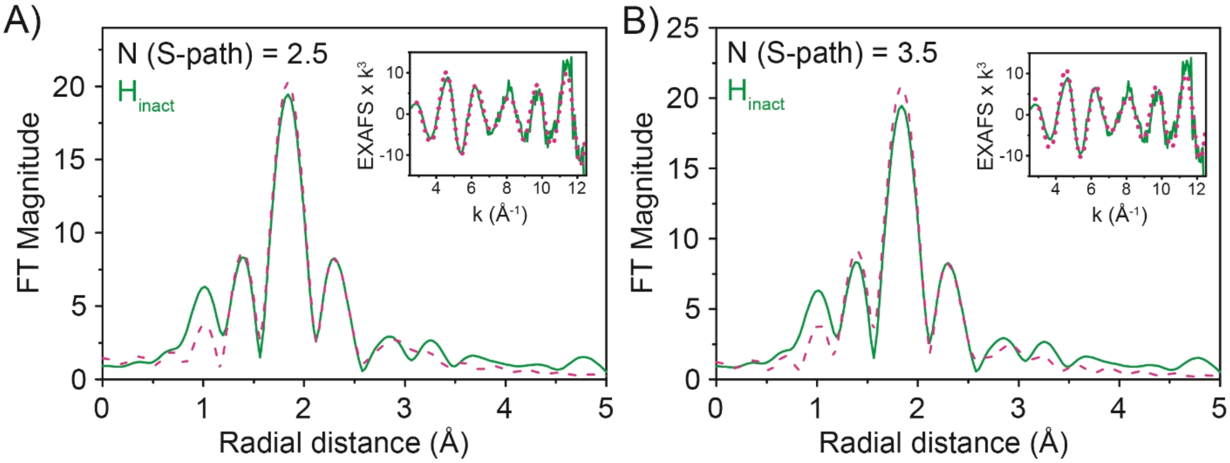

Fig. S14. k^3-EXAFS and FT spectra for the set of H_{inact} fits changing the degeneracy of the Fe-S path. **A**: decreasing the degeneracy (N) of the Fe-S path to N = 2.5 (Table S8). **B**: increasing the degeneracy (N) of the Fe-S path to 3.5 (Table S9).

Table S8. EXAFS fit parameters for *Dd*HydAB in the H_{inact} state with a degeneracy of the Fe-S path of N = 2.5 in Figure S14 A.

S_0^2= 0.9 / ΔE_0= 1.102 eV

R-factor: 0.0408

Scattering Path	N	σ^2 (x10^{-3} Å^2)	+/-	R (Å)	+/-
**Fe-C**	3	2.9	3.1	1.92513	0.0246
**Fe-C-O/N**	4	4.61	2.8	2.98123	0.028
**Fe-Fe**	1	2.00	set	2.62450	0.0215
**Fe-S**	**2.5**	-0.85	0. 6	2.23215	0.0055

Table S9. EXAFS fit parameters for *Dd*HydAB in the H_{inact} state with a degeneracy of the Fe-S path of N = 3.5 in Figure S14 B.

S_0^2= 0.9 / ΔE_0= 1.102 eV

R-factor: 0.0745

Scattering Path	N	σ^2 (x10^{-3} Å^2)	+/-	R (Å)	+/-
**Fe-C**	3	3.02	3.3	1.901	0.029
**Fe-C-O/N**	4	6.04	5.1	2.9891	0.044
**Fe-Fe**	1	2.00	set	2.638	0.028
**Fe-S**	**3.5**	1.28	0.8	2.233	0.008

As a validation of the apo-hydrogenase subtraction approach, we also decided to measure XANES and EXAFS on the diiron precursor complex used for artificial maturation of the apo-hydrogenase, referred to here as ADT, for which a high resolution crystal structure is available [2c]. The differences between this complex and $[2Fe]_H$ are *1)* ADT has an Fe(I)Fe(I) oxidation state, compared with Fe(II)Fe(I) in $[2Fe]_H$ in H_{ox} and Fe(II)Fe(II) in H_{inact}, *2)* ADT lacks the cysteine sulfur coordination present in $[2Fe]_H$, *3)* ADT contains an additional terminal CO ligand relative to $[2Fe]_H$, and *4)* ADT does not possess a bridging CO. Despite these differences the XANES (Figure S15) and EXAFS (Figure S16) spectra of ADT are surprisingly similar to the apo subtracted H_{ox} and H_{inact} spectra.

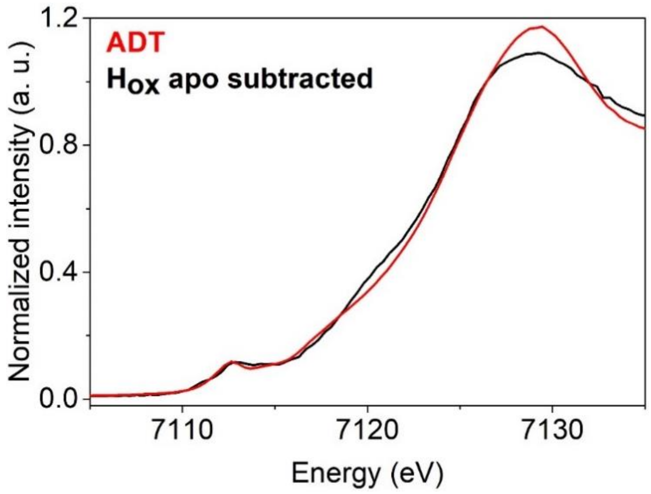

Fig. S15. Fe K-edge XANES spectra of H_{ox} apo-subtracted and ADT. The Fe K-edge XANES spectra of H_{ox} apo-subtracted (black trace) and ADT (red trace) are shown.

Attempts to fit the EXAFS data from ADT (Figure S16) demonstrated rather large uncertainties of the fitted parameters. The scattering paths are so strongly correlated with each other (Table S10) that the error associated with the fitted parameters is large (0.015 - 0.035 Å for the distances and 1.2 x 10^{-3} - 4.7 x 10^{-3} Å^2 for the DW factors). Fitting EXAFS data for such a complex system as the [FeFe] hydrogenase, where all the parameters are strongly correlated to each other, cannot give accurate values for all the parameters. Even though EXAFS analysis has its limitations, it does provide valuable information about the type of scatterers and their contribution to each state. The H_{inact} *vs* H_{ox} fits clearly show that more sulfur scatterers are present in the former.

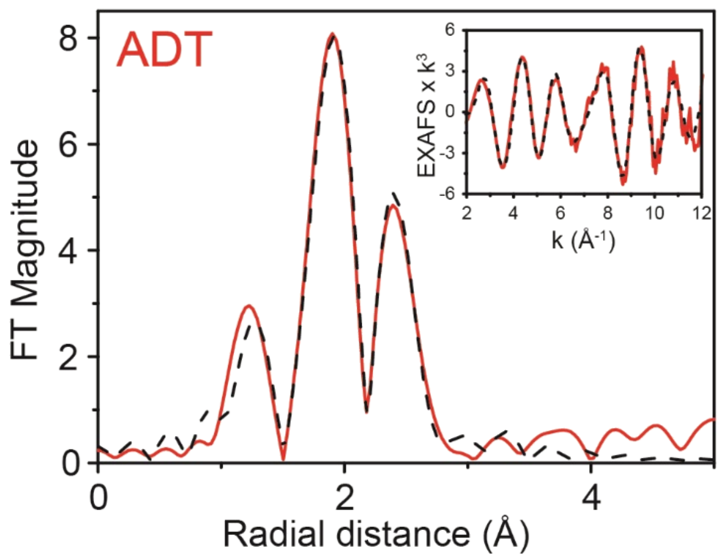

Fig. S16. *k*^{3}-EXAFS and FT spectra for the ADT fit (see Table S10 below)**.**

Table S10. EXAFS Fit Parameters for ADT.

S_0^2= 0.9 / ΔE_0= 1.079 eV

R-factor: 0.0118

Scattering Path	N	σ^2 (x10^{-3} Å^2)	+/-	R (Å)	+/-
**Fe-C^a**	2	4.77	1.6	1.824	0.015
**Fe-C^b**	1	3.37	4.7	2.081	0.034
**Fe-C-O/N**	6	11.19	1.7	3.037	0.012
**Fe-Fe**	1	5.77	2.27	2.447	0.022
**Fe-S**	2	2.66	1.2	2.345	0.012

TD-DFT Analysis of the pre-edge Fe XAS:

TD-DFT calculations were performed using the ωB97X functional (on top of TPSSh-optimized geometries) for H_{ox} and H_{inact}-SH QM/MM models and gave good agreement with the experimental Fe K-edge XAS spectra in the pre-edge region (Figure 4 in main article). Importantly, clear differences could be seen between the calculated spectra for H_{ox} and H_{inact}, which were also observable in the experimental spectra. According to our TD-DFT calculations, the first transition observed in the pre-edge of the experimental H_{ox} spectrum (which is not observed in the H_{inact} spectrum), corresponds to a transition into an empty low-lying d_{z^2} orbital, which is low in energy because of the unoccupied coordination site in H_{ox} (see Figure S17). At higher energy, the next pre-edge transitions, present in both H_{ox} and H_{inact}-SH models, correspond to excitations to the other "*d*-holes". The higher energy features of the pre-edge region in both models arise from metal ligand charge transfer (MLCT) transitions into bridging CO π^* orbitals. These MLCT transitions occur at the same energy for both metals in the $[2Fe]_H$ sub-cluster in the H_{inact}-SH model since it harbors a Fe(II)Fe(II) core. However, this feature is split in energy for H_{ox} since it has an Fe(I)Fe(II) core. The MLCT transitions occurring at Fe(I) take place at lower energy, explaining why the pre-edge of H_{ox} is broader than for H_{inact}. Other H_{inact} models with OH and Cl ligands were also calculated. The TD-DFT calculated XAS spectra in Figure S18 shows that both H_{inact}-Cl and H_{inact}-OH give almost identical spectra as H_{inact}-SH. The pre-edge XAS spectrum is thus not sensitive to the nature of the ligand. The positions of the MLCT transitions were found to be sensitive to the HF-exchange in the functional. The range-separated hybrid functional ωB97X (that has more correct long-range behavior and less self-interaction error) was found to give MLCT positions at slightly higher energy than the d-transitions that gave a spectrum in better agreement with experiment (see Figure S19).

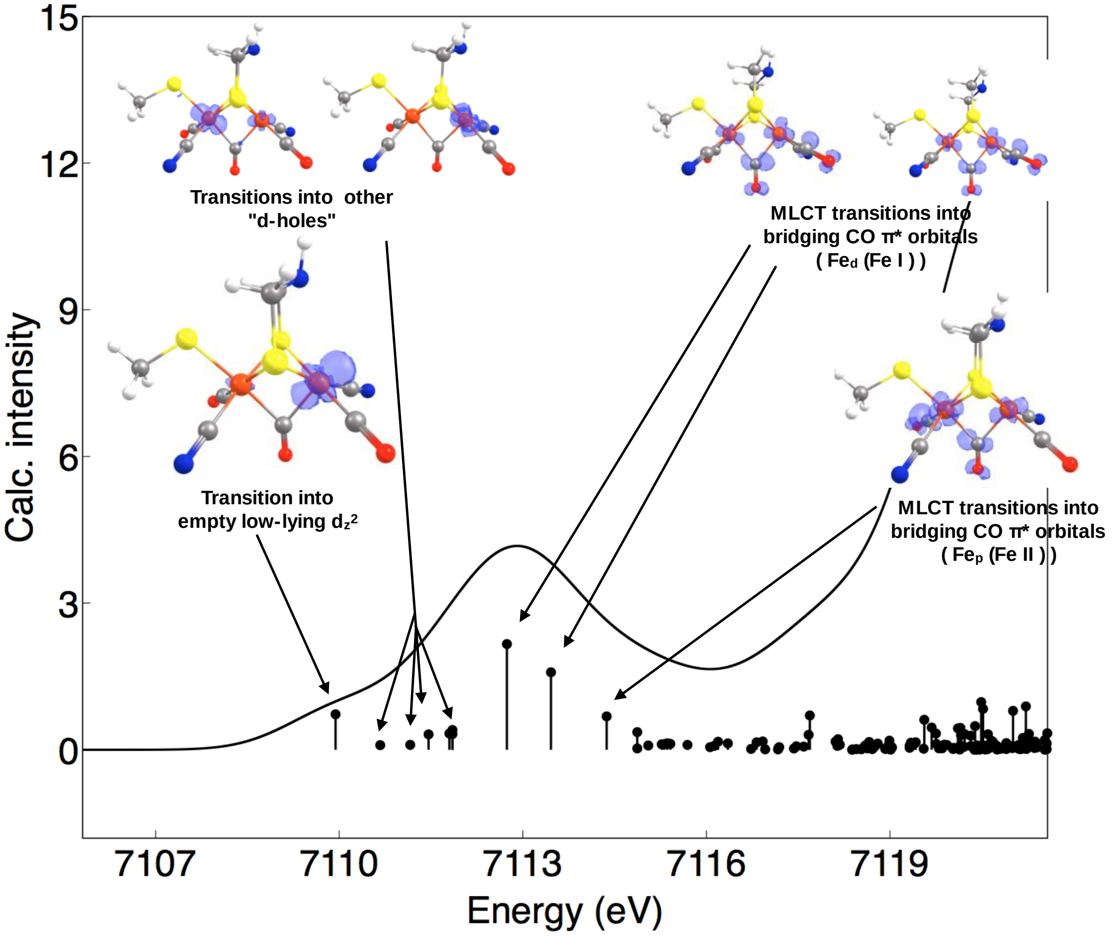

Fig. S17. TDDFT (ωB97X) difference densities of the electronic transitions in the Fe XAS calculation of H_{ox}. 2 eV FWHM broadening was applied based on fitting experimental pre-edges with blueprintXAS (parameters shown in Table S2) and an energy shift of 30.2 eV.

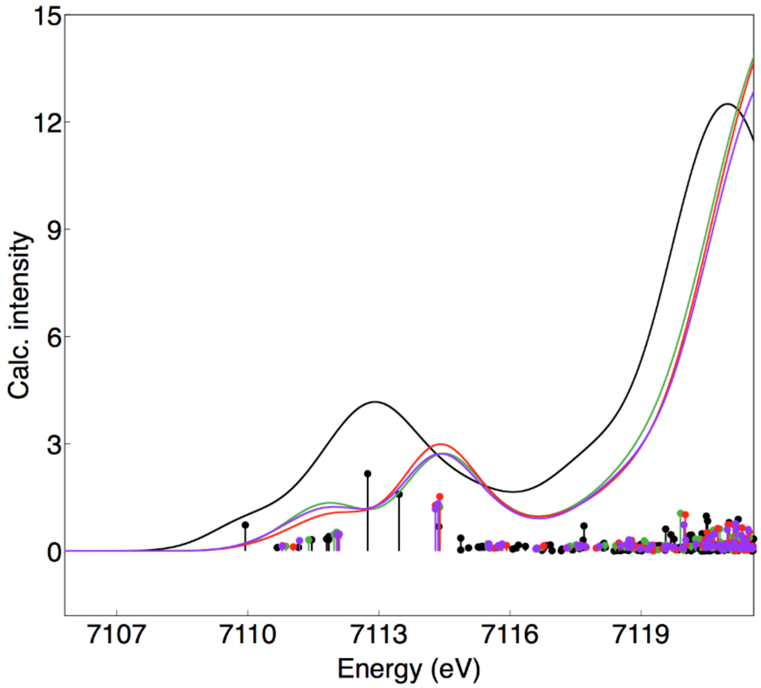

Fig. S18. TDDFT-calculated (ωB97X) Fe XAS pre-edge spectra of H_{ox} (black), H_{inact}-SH (green), H_{inact}-OH (purple) and H_{inact}-Cl (red) models. 2 eV FWHM broadening was applied based on fitting experimental pre-edges with blueprintXAS (parameters shown in Tables S2 and 3) and an energy shift of 30.2 eV.

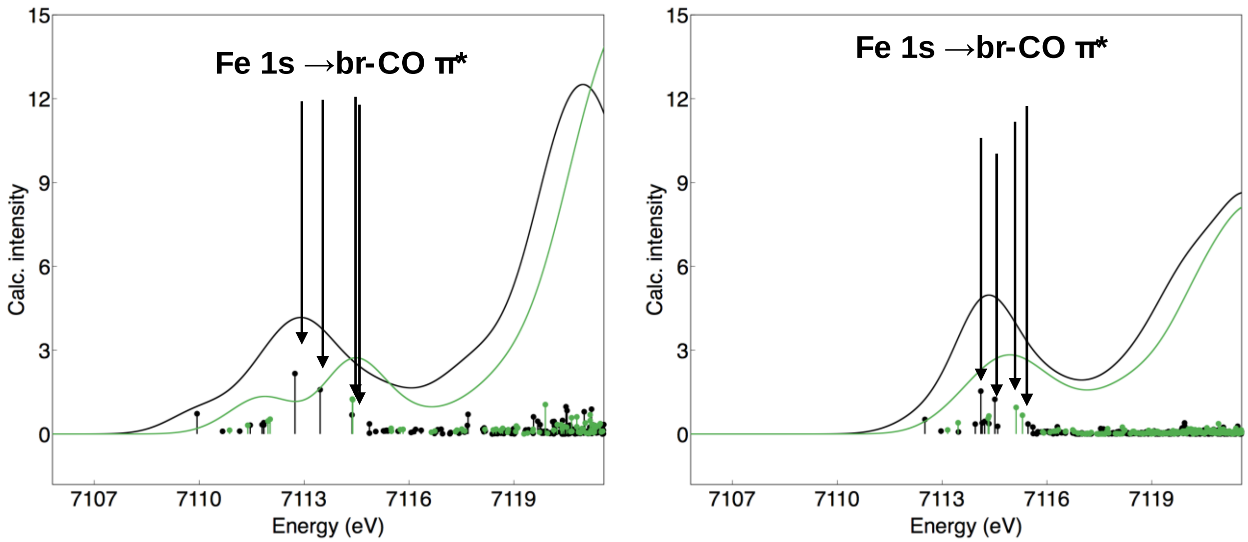

Fig. S19. Comparison of TDDFT-calculated pre-edges with ωB97X (left) and TPSSh (right) for H_{ox} (black) and H_{inact} (green) models. As can be seen all transition energies are shifted towards higher energy with TPSSh than ωB97X but overall the same pre-edge transitions are present in both spectra. The relative positions of the most intense transitions are Fe 1s → br-CO π* charge-transfer transitions are especially sensitive to the HF exchange percentage in the functional. 2 eV FWHM broadening was applied based on fitting experimental pre-edges with blueprintXAS (parameters shown in Tables S2 and 3) and an energy shift of 30.2 eV.

Vibrational Spectroscopy Supplementary Discussion.

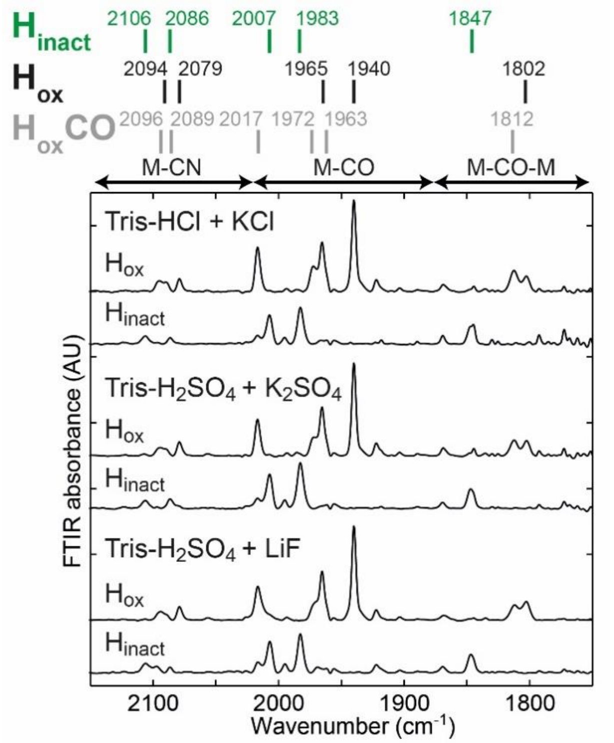

Fig. S20. IR spectra of H_{inact} prepared in the presence and absence of chloride. *Dd*HydAB (200 μM) in 50 mM Tris-HCl, 500 mM KCl, pH 8 (top two spectra); 50 mM Tris-H_2SO_4, 500 mM K_2SO_4, pH 8 (middle two spectra); or 50 mM Tris-H_2SO_4, 500 mM LiF, pH 8 (bottom two spectra) was oxidized under N_2 with 1 mM thionine acetate (H_{ox} spectra). This was followed by addition of 10 mM Na_2S and 10 mM thionine acetate, and exposure to air (H_{inact} spectra). In each case, the H_{inact} state could be formed indicating that the presence of chloride is not required for H_{inact} formation. Some of the H_{ox}-CO state (from H-cluster decomposition) contributes to the H_{ox} spectra.

Table S11. Table of experimental and calculated IR frequencies (cm^{-1}) of the diatomic ligands for H_{ox} and H_{inact}. A comparison of the experimental IR frequencies for the H_{ox} and H_{inact} states and the calculated values for the H_{ox}, H_{inact}-SH, H_{inact}-OH and H_{inact}-Cl models as described in the QM-MM calculations section is presented**.**

Ligand	H_{ox} exp	H_{ox} model	H_{inact} exp	H_{inact}-SH model	H_{inact}-OH model	H_{inact}-Cl model
CN_t^1	2094	2166.4	2106	2170.1	2170.7	2172.2
CN_t^2	2079	2140.0	2086	2160.8	2166.6	2168.5
CO_t^1	1965	2035.8	2007	2058.2	2060.4	2084.7
CO_t^2	1940	2012.2	1983	2023.1	2023.9	2028.0
CO_b	1802	1890.3	1847	1919.0	1919.0	1924.2

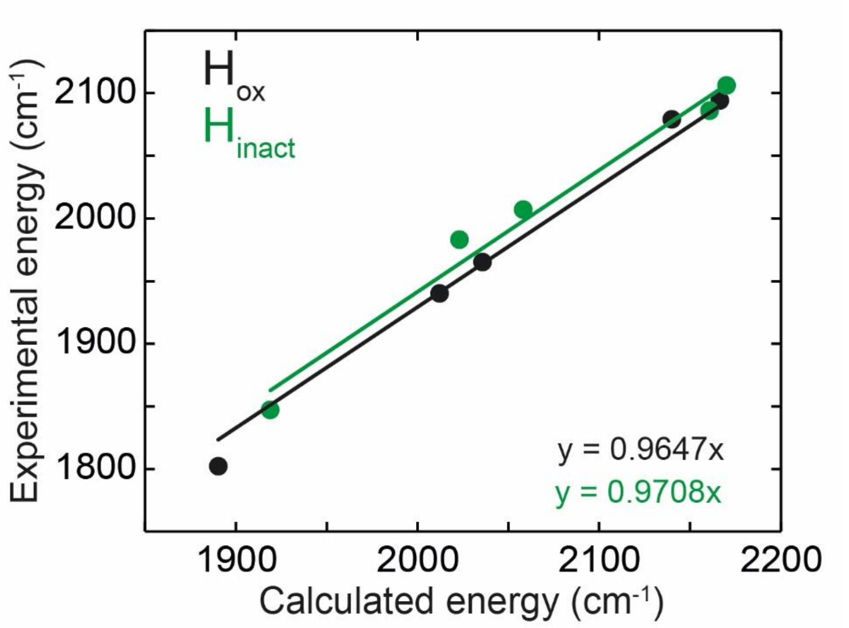

Fig. S21. Correlation of the experimental and calculated IR frequencies for H$_{ox}$ and H$_{inact}$. Experimental FTIR frequencies for the H$_{ox}$ and H$_{inact}$ states (y-axis) are plotted against the calculated frequencies (x-axis) using the H$_{ox}$ (black circles) and H$_{inact}$-SH (green circles) models described in the QM/MM calculations section. Regression lines are plotted with an x-axis intercept at y = 0, and the formulae of the regression lines are shown on the chart. A value of 0.964 was taken as the scaling factor for the calculated frequencies used to derive the spectra presented in the main text and Figure S22.

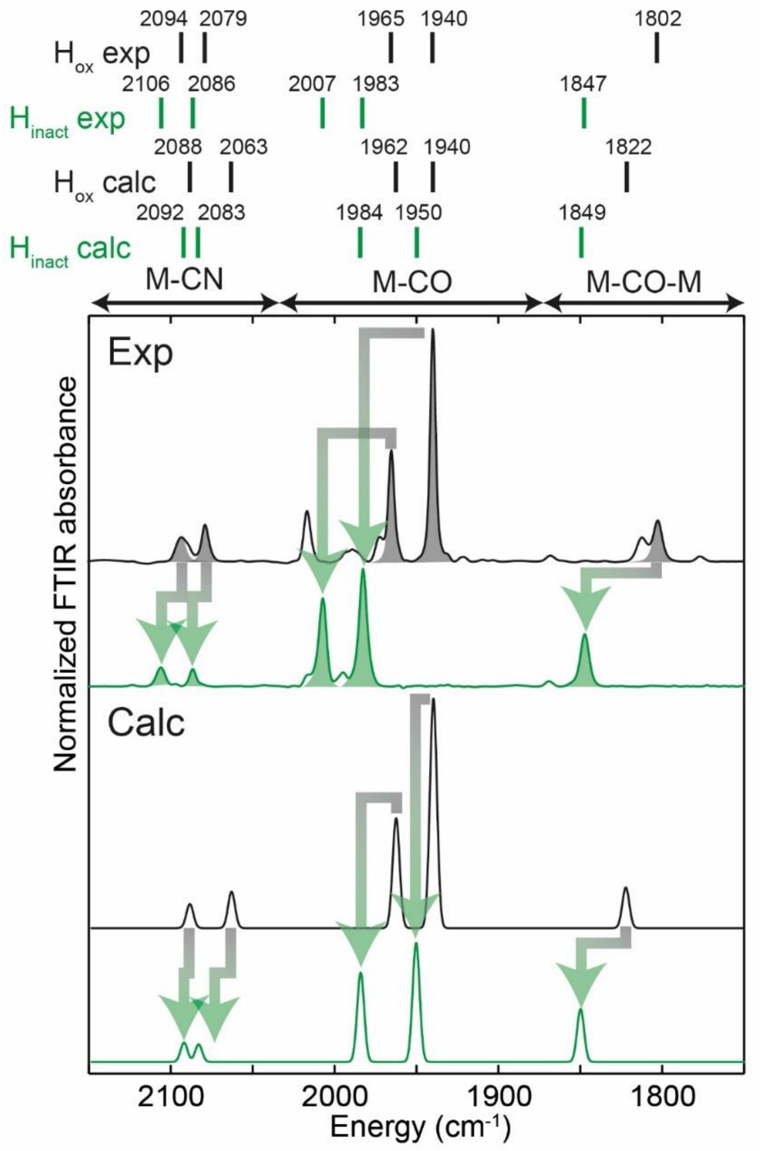

Fig. S22. Alternative view of the experimental and calculated IR spectra of H_{ox} and H_{inact}. The experimental and calculated IR spectra of H_{ox} and H_{inact} are presented with H_{ox} and H_{inact} vertically aligned to highlight the shifts in the IR bands when going from H_{ox} to H_{inact} and how well the calculations capture this behavior. Calculated spectra have been generated using frequencies scaled by a factor of 0.964. Frequencies have been convoluted with Voigtian line broadening with a 15 cm^{-1} FWHM and intensities are scaled empirically.

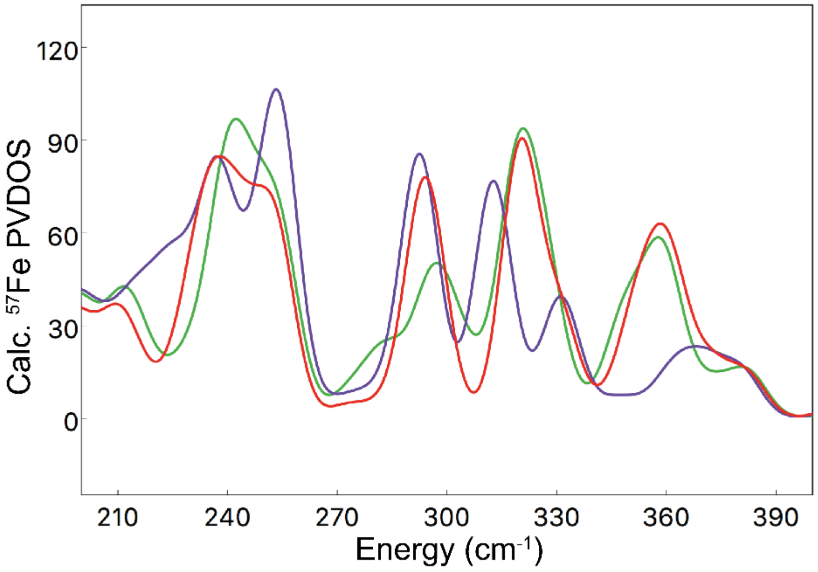

Fig. S23. QM/MM-calculated NRVS spectra (200-500 cm^{-1} region) of H_{inact}-SH (green), H_{inact}-OH (purple) and H_{inact}-Cl (red) models.

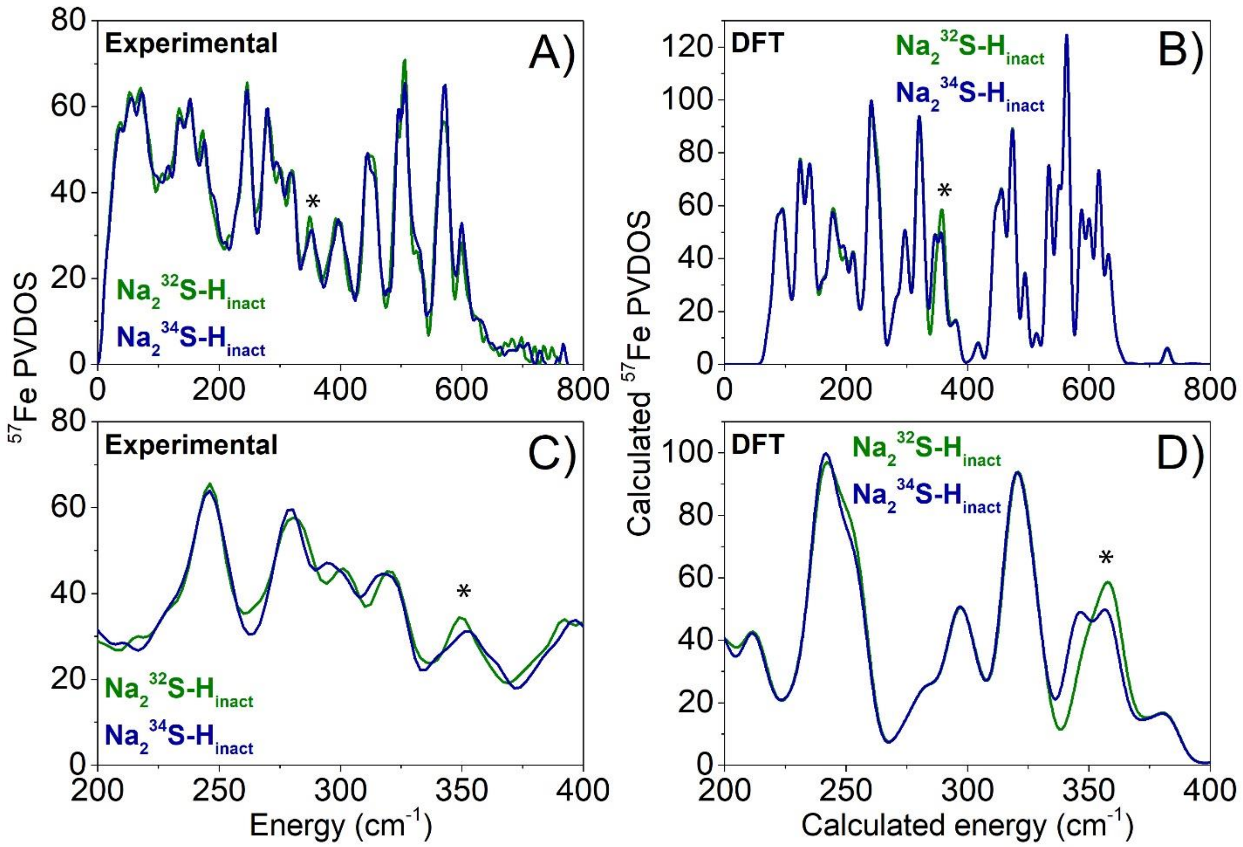

Fig. S24. NRVS experimental and QM/MM-calculated ^{57}Fe NRVS spectra of *Dd*HydAB in the H_{inact} state with ^{32}S (green) and ^{34}S (royal blue) ligand. Spectra **A** (full spectrum) and **C** (close-up view of the Fe-S region) represent the experimental spectra of H_{inact} prepared in solution with $Na_2{}^{32}S$ (green trace) *vs* H_{inact} prepared in solution with $Na_2{}^{34}S$ (royal blue trace). Spectrum **B** (full spectrum) and **D** (close-up view of the Fe-S region) represent the QM/MM-calculated ^{57}Fe NRVS spectra of the H_{inact}-SH model with a ^{32}S (green trace) and ^{34}S (royal blue trace) substituted SH-ligand.

The close-up view of the calculated spectra shows a shift to lower energy of the peak at ≈ 350 cm^{-1} (marked with an asterisk) when going from ^{32}S (green) to ^{34}S (royal blue) as it should for a heavier atom. This results in the broad green peak (^{32}S, asterisk) splitting into two blue peaks (^{34}S), or an overall intensity reduction. The calculated isotope shifts are also shown in Table S12. The experimental spectrum is consistent with the intensity reduction and the shoulder at ≈ 350 cm^{-1} (asterisk) is also shifted toward lower energy. Since the shifts are very subtle, it is difficult to observe them in the experimental spectra because they are within the noise.

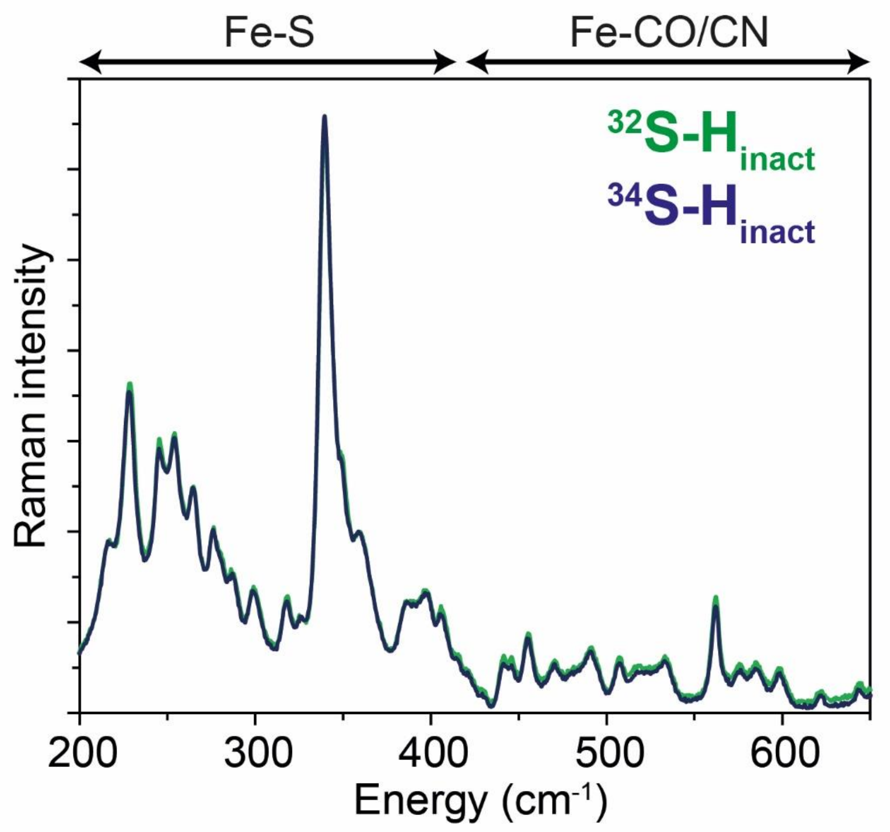

Fig. S25. Resonance Raman spectra of *Dd*HydAB in the H_{inact} state. Resonance Raman spectra were measured on 3 mM *Dd*HydAB samples at 80 K using 514 nm excitation. Experimental spectra of H_{inact} prepared with natural abundance Na_2S (green trace) and ^{34}S-labelled Na_2S. The spectra are normalized to modes at 622 cm^{-1} and 644 cm^{-1} corresponding to the amino acid side chains phenylalanine and tyrosine, respectively.[39] Calculated resonance Raman band positions using the H_{inact}-SH QM/MM model with ^{32}S and ^{34}S bound at open coordination site of Fe_d are presented in Table S12 and shown as Movies S1 and S2.

Table S12. Table of the calculated vibrational frequencies (cm^{-1}) of the modes in the Fe-S region with the largest SH group contribution, with ^{32}S and ^{34}S substitution in H_{inact}. Fe mass is 55.8 amu or 57 amu (values in parentheses).

[2Fe]-^{32}SH	[2Fe]-^{34}SH	Δ
**348.6 (348.3)**	346.0 (345.5)	2.6 (2.8)
**359.9 (359.5)**	358.8 (358.7)	1.1 (0.8)

*These specific vibrational modes can be visualized as separate MPEG movie files.

Possible H_{inact} Formation Mechanism

We note that it is not possible to provide accurate ΔG values in the last two steps of the mechanism. The reliable accurate calculation of redox potentials and protonation/deprotonation reaction energies is very difficult in computational chemistry due to the big environmental effect difference between the two charged states.

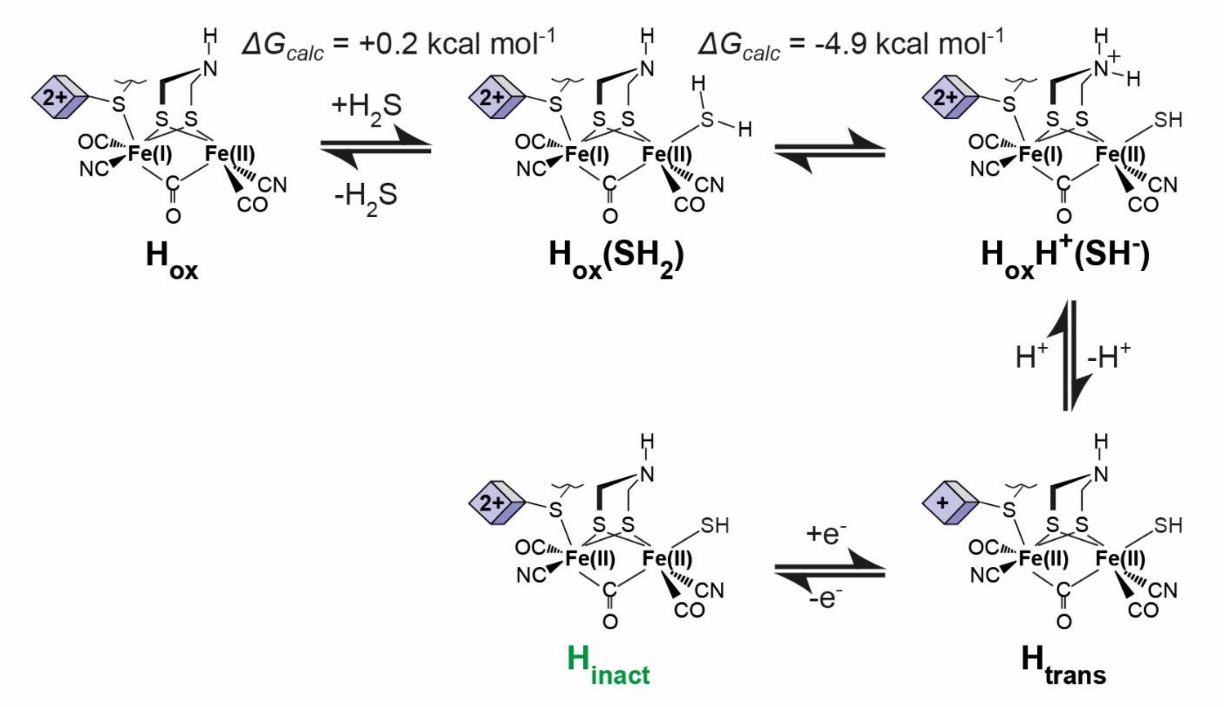

Fig. S26. Proposed scheme for H_{inact} formation from H_{ox}. First H_2S binds to the apical coordination site on Fe_d in the H_{ox} state forming $H_{ox}(SH_2)$. Binding has been calculated to be thermoneutral with an estimated ΔG of +0.2 kcal mol^{-1}. This is followed by proton transfer to the nitrogen base of the ADT bridge giving $H_{ox}H^+(SH^-)$. This step is calculated to be thermodynamically favorable with an estimated ΔG of -4.9 kcal mol^{-1}. Deprotonation of the ADT bridge via the proton transfer channel leads to electron transfer from $[2Fe]_H$ to $[4Fe\text{-}4S]_H$, yielding the spectroscopically characterized H_{trans} state. Finally, H_{trans} is converted to H_{inact} by oxidation of $[4Fe\text{-}4S]_H$. An alternative proposal of H_2O binding gives a binding energy of ΔG= -6 kcal/mol but an H_2O ligand cannot be deprotonated by the ADT ligand (attempted optimizations resulted in spontaneous proton transfer back), unlike an H_2S ligand.

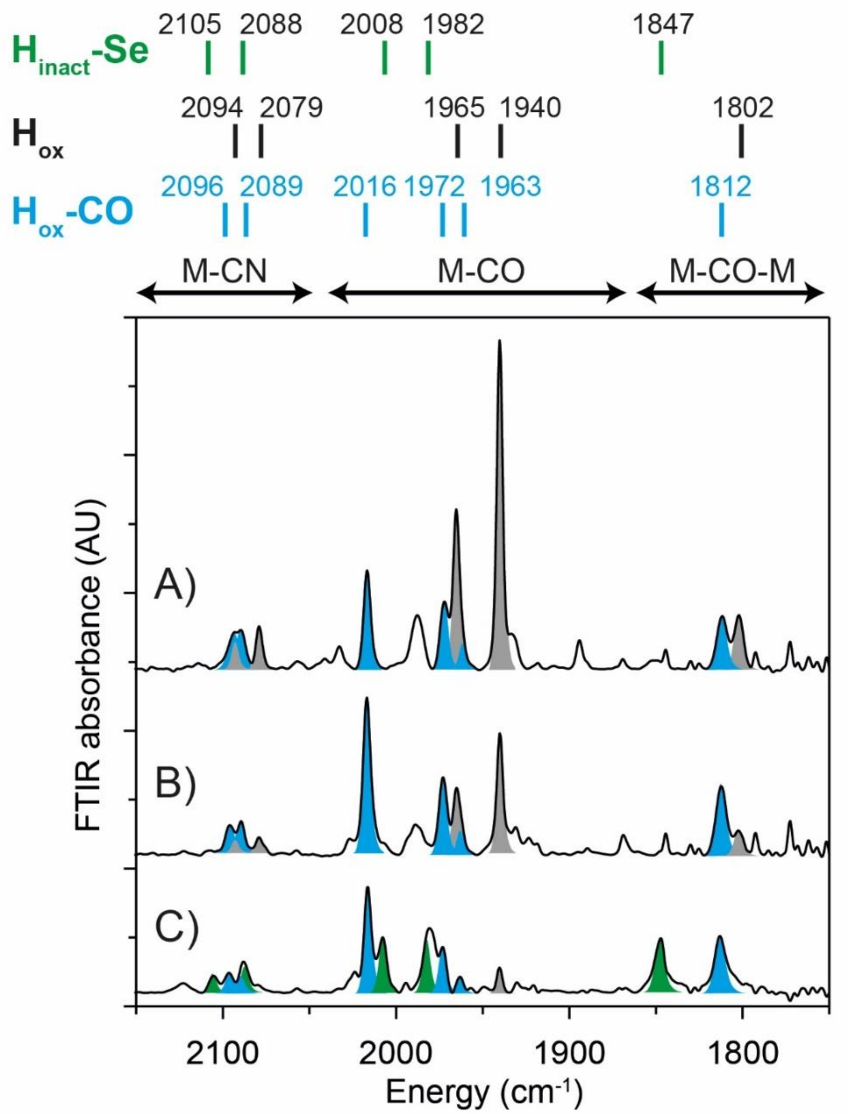

Fig. S27. IR spectra illustrate the formation of H$_{inact}$ with Na_2Se. A) *Dd*HydAB under an N_2 atmosphere in 25 mM Tris-HCl, 25 mM KCl, pH 8 with 10 mM Na_2Se. B) The same as in A but with addition of 10 mM hexaammineruthenium (III) chloride. C) The same as in B except *Dd*HydAB was first exchanged into 50 mM sodium acetate pH 4.

Captions to Supplementary movie files

Movie S1. MPEG file showing the animated motion corresponding to the 348.6 cm^{-1} (^{32}S) and 346.0 cm^{-1} (^{34}S) vibrational modes. The mode is derived from QM/MM calculations of the H$_{inact}$-SH model.

Movie S2. MPEG file showing the animated motion corresponding to the 359.9 cm^{-1} (^{32}S) and 358.8 cm^{-1} (^{34}S) vibrational modes. The mode is derived from QM/MM calculations of the H$_{inact}$-SH model.

Supplementary References

[1] a) J. A. Birrell, K. Wrede, K. Pawlak, P. Rodriguez-Maciá, O. Rüdiger, E. J. Reijerse, W. Lubitz, *Isr. J. Chem.* **2016**, *56*, 852-863; b) P. Rodríguez-Maciá, E. J. Reijerse, M. van Gastel, S. DeBeer, W. Lubitz, O. Rüdiger, J. A. Birrell, *J. Am. Chem. Soc.* **2018**, *140*, 9346-9350.

[2] a) R. Gilbert-Wilson, J. F. Siebel, A. Adamska-Venkatesh, C. C. Pham, E. Reijerse, H. Wang, S. P. Cramer, W. Lubitz, T. B. Rauchfuss, *J. Am. Chem. Soc.* **2015**, *137*, 8998-9005; b) A. Le Cloirec, S. P. Best, S. Borg, S. C. Davies, D. J. Evans, D. L. Hughes, C. J. Pickett, *Chem. Commun.* **1999**, 2285-2286; c) H. Li, T. B. Rauchfuss, *J. Am. Chem. Soc.* **2002**, *124*, 726-727.

[3] W. Kabsch, *Acta Crystallogr. D* **2010**, *66*, 125-132.

[4] M. D. Winn, C. C. Ballard, K. D. Cowtan, E. J. Dodson, P. Emsley, P. R. Evans, R. M. Keegan, E. B. Krissinel, A. G. W. Leslie, A. McCoy, S. J. McNicholas, G. N. Murshudov, N. S. Pannu, E. A. Potterton, H. R. Powell, R. J. Read, A. Vagin, K. S. Wilson, *Acta Crystallogr. D* **2011**, *67*, 235-242.

[5] P. R. Evans, G. N. Murshudov, *Acta Crystallogr. D* **2013**, *69*, 1204-1214.

[6] A. J. McCoy, R. W. Grosse-Kunstleve, P. D. Adams, M. D. Winn, L. C. Storoni, R. J. Read, *J. Appl. Crystallogr.* **2007**, *40*, 658-674.

[7] G. N. Murshudov, A. A. Vagin, E. J. Dodson, *Acta Crystallogr. D* **1997**, *53*, 240-255.

[8] V. B. Chen, W. B. Arendall, 3rd, J. J. Headd, D. A. Keedy, R. M. Immormino, G. J. Kapral, L. W. Murray, J. S. Richardson, D. C. Richardson, *Acta Crystallogr. D* **2010**, *66*, 12-21.

[9] L. Ten, *Acta Crystallogr. A***1973**, *29*, 183-191.

[10] Y. Ilina, C. Lorent, S. Katz, J.-H. Jeoung, S. Shima, M. Horch, I. Zebger, H. Dobbek, *Angew. Chem. Int. Ed.* **2019***, 58,* 18710-18714.

[11] J. B. H. Strautmann, S. D. George, E. Bothe, E. Bill, T. Weyhermüller, A. Stammler, H. Bögge, T. Glaser, *Inorg. Chem.* **2008**, *47*, 6804-6824.

[12] B. Ravel, M. Newville, *J. Synchrotron Rad.* **2005**, *12*, 537-541.

[13] S. I. Zabinsky, J. J. Rehr, A. Ankudinov, R. C. Albers, M. J. Eller, *Phys. Rev. B* **1995**, *52*, 2995-3009.

[14] Y. Nicolet, C. Piras, P. Legrand, C. E. Hatchikian, J. C. Fontecilla-Camps, *Structure* **1999**, *7*, 13-23.

[15] a) S. Metz, J. Kästner, A. A. Sokol, T. W. Keal, P. Sherwood, *Wiley Interdiscip. Rev. Comput. Mol. Sci.* **2014**, *4*, 101-110; b) P. Sherwood, A. H. de Vries, M. F. Guest, G. Schreckenbach, C. R. A. Catlow, S. A. French, A. A. Sokol, S. T. Bromley, W. Thiel, A. J. Turner, S. Billeter, F. Terstegen, S. Thiel, J. Kendrick, S. C. Rogers, J. Casci, M. Watson, F. King, E. Karlsen, M. Sjøvoll, A. Fahmi, A. Schäfer, C. Lennartz, *J. Mol. Struc. THEOCHEM* **2003**, *632*, 1-28.

[16] a) F. Neese, *Wiley Interdiscip. Rev. Comput. Mol. Sci.* **2012**, *2*, 73-78; b) F. Neese, *Wiley Interdiscip. Rev. Comput. Mol. Sci.* **2018**, *8*, e1327.

[17] W. Sturhahn, *Hyperfine Interact.* **2000**, *125*, 149-172.

[18] J. Esselborn, N. Muraki, K. Klein, V. Engelbrecht, N. Metzler-Nolte, U. P. Apfel, E. Hofmann, G. Kurisu, T. Happe, *Chem. Sci.* **2016**, *7*, 959-968.

[19] J. Duan, M. Senger, J. Esselborn, V. Engelbrecht, F. Wittkamp, U.-P. Apfel, E. Hofmann, S. T. Stripp, T. Happe, M. Winkler, *Nat. Commun.* **2018**, *9*, 4726.

[20] R. B. Best, X. Zhu, J. Shim, P. E. Lopes, J. Mittal, M. Feig, A. D. Mackerell, Jr., *J. Chem. Theory Comput.* **2012**, *8*, 3257-3273.

[21] a) A. D. Becke, *Phys. Rev. A Gen. Phys.* **1988**, *38*, 3098-3100; b) J. P. Perdew, *Phys. Rev. B Condens. Matter* **1986**, *33*, 8822-8824.

[22] F. Weigend, R. Ahlrichs, *Phys. Chem. Chem. Phys.* **2005**, *7*, 3297-3305.

[23] A. E. W. Reed, R. B.; Weinhold, F., *J. Chem. Phys.* **1985**, *83*, 735-746.

[24] a) M. J. Abraham, T. Murtola, R. Schulz, S. Páll, J. C. Smith, B. Hess, E. Lindahl, *SoftwareX* **2015**, *1-2*, 19-25; b) B. Hess, C. Kutzner, D. van der Spoel, E. Lindahl, *J. Chem. Theory Comput.* **2008**, *4*, 435-447; c) S. Pronk, S. Pall, R. Schulz, P. Larsson, P. Bjelkmar, R. Apostolov, M. R. Shirts, J. C. Smith, P. M. Kasson, D. van der Spoel, B. Hess, E. Lindahl, *Bioinformatics* **2013**, *29*, 845-854.

[25] a) B. Hess, *J. Chem. Theory Comput.* **2008**, *4*, 116-122; b) B. Hess, H. Bekker, H. J. C. Berendsen, J. G. E. M. Fraaije, *J. Comput. Chem.* **1997**, *18*, 1463-1472.

[26] a) G. J. K. Martyna, M. L.; Tuckerman, M., *J. Chem. Phys.* **1992**, *97*, 2635-2643; b) S. Nosé, *J. Chem. Phys.* **1984**, *81*, 511-519; c) S. Nosé, *Mol. Phys.* **1984**, *52*, 255-268; d) W. G. Hoover, *Phys. Rev. A Gen. Phys.* **1985**, *31*, 1695-1697.

[27] B. Benediktsson, R. Bjornsson, *Inorg Chem* **2017**, *56*, 13417-13429.

[28] P. Sherwood, in *Modern Methods and Algorithms of Quantum Chemistry Proceedings* (Ed.: J. Grotendorst), John von Neumann Institute for Computing : Jülich, Germany, pp. 285-305.

[29] a) V. N. S. Staroverov, G. E.; Tao, J.; Perdew, J. P., *J. Chem. Phys.* **2003**, *119*, 12129-12137; b) J. Tao, J. P. Perdew, V. N. Staroverov, G. E. Scuseria, *Phys. Rev. Lett.* **2003**, *91*, 146401.

[30] a) S. Grimme, J. Antony, S. Ehrlich, H. Krieg, *J. Chem. Phys.* **2010**, *132*, 154104; b) S. Grimme, S. Ehrlich, L. Goerigk, *J. Comput. Chem.* **2011**, *32*, 1456-1465.

[31] a) E. B. van Lenthe, E. J.; Snijders, J. G., *J. Chem. Phys.* **1993**, *99*, 4597-4610; b) C. van Wüllen, *J. Chem. Phys.* **1998**, *109*, 392.

[32] D. A. Pantazis, X. Y. Chen, C. R. Landis, F. Neese, *J. Chem. Theory Comput.* **2008**, *4*, 908-919.

[33] a) R. N. Izsák, F., *J. Chem. Phys.* **2011**, *135*, 144105; b) F. Neese, F. Wennmohs, A. Hansen, U. Becker, *Chem. Phys.* **2009**, *356*, 98-109.

[34] W. Smith, T. R. Forester, *J. Mol. Graph.* **1996**, *14*, 136-141.

[35] J. Kastner, J. M. Carr, T. W. Keal, W. Thiel, A. Wander, P. Sherwood, *J. Phys. Chem. A* **2009**, *113*, 11856-11865.

[36] L. S. Kau, D. J. Spira-Solomon, J. E. Penner-Hahn, K. O. Hodgson, E. I. Solomon, *J. Am. Chem. Soc.* **1987**, *109*, 6433-6442.

[37] C. Van Stappen, R. Davydov, Z.-Y. Yang, R. Fan, Y. Guo, E. Bill, L. C. Seefeldt, B. M. Hoffman, S. DeBeer, *Inorg. Chem.* **2019**, *58*, 12365-12376.
[38] a) J. M. Guss, H. D. Bartunik, H. C. Freeman, *Acta Crystallogr. B* **1992**, *48*, 790-811; bE. I. Solomon, R. K. Szilagyi, S. DeBeer George, L. Basumallick, *Chem. Rev.* **2004**, *104*, 419-458.
[39] F. M. B. Paulo T.C. Freire, José A. Lima, Francisco E.A. Melo and Josué Mendes Filho, in *Raman Spectroscopy and Applications,, Vol. Open access peer-reviewed Edited Volume*, IntechOpen, **2017**.
